# Transaldolase inhibition impairs mitochondrial respiration and induces a starvation-like longevity response in *Caenorhabditis elegans*

**DOI:** 10.1371/journal.pgen.1006695

**Published:** 2017-03-29

**Authors:** Christopher F. Bennett, Jane J. Kwon, Christine Chen, Joshua Russell, Kathlyn Acosta, Nikolay Burnaevskiy, Matthew M. Crane, Alessandro Bitto, Helen Vander Wende, Marissa Simko, Victor Pineda, Ryan Rossner, Brian M. Wasko, Haeri Choi, Shiwen Chen, Shirley Park, Gholamali Jafari, Bryan Sands, Carissa Perez Olsen, Alexander R. Mendenhall, Philip G. Morgan, Matt Kaeberlein

**Affiliations:** 1 Department of Pathology, University of Washington, Seattle, WA, United States of America; 2 Molecular and Cellular Biology Program, University of Washington, Seattle, WA, United States of America; 3 Molecular Medicine and Mechanisms of Disease Program, University of Washington, Seattle, WA, United States of America; 4 Division of Basic Sciences, Fred Hutchinson Cancer Research Center, Seattle, WA, United States of America; 5 Center for Integrated Brain Research, Seattle Children’s Research Institute, Seattle, WA, United States of America; 6 Department of Anesthesiology, University of Washington School of Medicine, Seattle, WA, United States of America; Princeton, UNITED STATES

## Abstract

Mitochondrial dysfunction can increase oxidative stress and extend lifespan in *Caenorhabditis elegans*. Homeostatic mechanisms exist to cope with disruptions to mitochondrial function that promote cellular health and organismal longevity. Previously, we determined that decreased expression of the cytosolic pentose phosphate pathway (PPP) enzyme transaldolase activates the mitochondrial unfolded protein response (UPR^mt^) and extends lifespan. Here we report that transaldolase (*tald-1*) deficiency impairs mitochondrial function *in vivo*, as evidenced by altered mitochondrial morphology, decreased respiration, and increased cellular H_2_O_2_ levels. Lifespan extension from knockdown of *tald-1* is associated with an oxidative stress response involving p38 and c-Jun N-terminal kinase (JNK) MAPKs and a starvation-like response regulated by the transcription factor EB (TFEB) homolog HLH-30. The latter response promotes autophagy and increases expression of the flavin-containing monooxygenase 2 (*fmo-2*). We conclude that cytosolic redox established through the PPP is a key regulator of mitochondrial function and defines a new mechanism for mitochondrial regulation of longevity.

## Author summary

There are a growing number of studies linking mitochondrial dysfunction to enhanced longevity, especially in the nematode *C*. *elegans*. The reasons for these pro-longevity effects have been elusive, but one current model is that adaptive responses to mitochondrial inhibition promote organismal health and stress resistance. Here, we report an intriguing example of mitochondrial stress induced by inhibition of a cytosolic metabolic pathway that extends lifespan in worms. We find that inhibition of the pentose phosphate pathway, which is essential for cytosolic redox homeostasis, affects multiple parameters of mitochondrial function and activates a starvation-like response that promotes longevity through recycling of damaged cellular components and induction of the enzyme flavin-containing monooxygenase 2. These results establish novel links between the pentose phosphate pathway, mitochondrial function, redox homeostasis, and organismal aging.

## Introduction

Mitochondria are the primary sites of aerobic metabolism and energy production in the cell. The mitochondrial free radical theory of aging posits that reactive oxygen species (ROS) produced by mitochondria during oxidative metabolism cause damage to macromolecules which, over time, leads to the accumulation of cellular, tissue, and organismal declines, and ultimately death [1, 2]. In general, mitochondrial dysfunction is detrimental, and has been causally implicated in several age-related diseases, as well as severe, early-onset mitochondrial disorders. Paradoxically, however, inhibition of mitochondrial function has, in some cases, been associated with increased longevity in laboratory organisms from yeast to mammals [3]. This is particularly evident in *C*. *elegans*, where inhibition of mitochondrial respiration by mutation or knockdown of numerous electron transport chain (ETC) components usually increases lifespan [4, 5]. Mild oxidative stress can also increase lifespan [3], perhaps by inducing adaptive responses that compensate for these insults and provide cytoprotective effects to improve cellular stress resistance [6].

The mechanistic basis for lifespan extension in response to mitochondrial inhibition and mild oxidative stress in *C*. *elegans* is an active area of investigation. One mitochondrial stress pathway that has been associated with worm longevity in this context is the mitochondrial unfolded protein response (UPR^mt^) [7–9]. The UPR^mt^ is a coordinated response to mitochondrial stress resulting in upregulation of mitochondrial chaperones, import machinery, and proteases, while negatively regulating expression of nuclear- and mitochondrial-encoded ETC components [10–12]. Activation of the UPR^mt^ is regulated by the ATFS-1 transcription factor, which translocates to the nucleus in response to mitochondrial stress and directly activates transcription of several UPR^mt^ target genes [11, 13]. Whether the UPR^mt^ plays a direct role in determining longevity remains unclear. Lifespan extension by ETC inhibition or treatment with the ROS-generating compound paraquat is correlated with induction of the UPR^mt^ [7, 10, 14]; however, deletion or RNAi knockdown of *atfs-1* blocks induction of several UPR^mt^ target genes but does not prevent or attenuate lifespan extension following inhibition of the ETC [15, 16]. Similarly, constitutive active alleles of *atfs-1* cause activation of the UPR^mt^ but do not extend lifespan [15–17].

There is experimental evidence supporting a role for several factors other than the UPR^mt^ in lifespan extension downstream of mitochondrial inhibition in *C*. *elegans*, including the hypoxic response transcription factor HIF-1, CEP-1/p53, the CEH-23 transcription factor, components of the intrinsic apoptotic pathway, and the p38 MAPK PMK-3 [18–22]. A majority of these studies have been performed using mutants with defective ETC function, such as the Rieske iron-sulfur protein gene *isp-1(qm150)* allele and the ubiquinone biosynthetic gene *clk-1(qm30)* allele. With the possible exception of *pmk-3*, none of these factors is able to account for the full lifespan extension following RNAi knockdown of ETC genes such as the cytochrome c oxidase gene *cco-1*. This is consistent with a model proposed by the Hekimi lab that RNAi inhibition of ETC function promotes worm longevity by a mechanism distinct from mutations that impair ETC function [23].

Uncovering the genetic pathways and molecular mechanisms by which mitochondria influence aging and disease is critical both for developing better models of biological aging, as well as for identifying interventions to promote health and longevity. As mentioned above, low levels of oxidative stress can be beneficial to cellular health, but high levels can cause irreparable damage. This biphasic or non-linear relationship between mitochondrial ROS and survival is commonly referred to as mitohormesis, and posits that ROS act as signaling molecules to induce adaptive mechanisms [6]. This has been observed in *C*. *elegans*, where different levels of RNAi knockdown of a single mitochondrial gene can cause differential effects on lifespan and other physiological markers [24, 25]. The beneficial hormetic effects associated with elevated ROS are due to the contribution of multiple protective responses that are still being discovered. Therefore, we sought to identify and determine the interconnectivity of novel longevity pathways distinct from the UPR^mt^ that are engaged by oxidative and mitochondrial stress.

Although the UPR^mt^ does not appear to directly mediate lifespan extension, we reasoned that the partial correlation between activation of the UPR^mt^ and longevity could be used to identify novel factors and mechanisms of action within the mitochondrial longevity network. To identify such factors, we performed a genome-wide RNAi screen for *C*. *elegans* genes that negatively regulate the UPR^mt^ by looking for RNAi clones that activated the UPR^mt^ reporter *hsp-6p*::*gfp* [15]. Some, but not all, of these genes also negatively affected lifespan such that RNAi knockdown increased longevity. One such gene is *tald-1*, which encodes the pentose phosphate pathway (PPP) enzyme transaldolase. The PPP pathway is a cytosolic metabolic pathway that functions to produce NADPH, ribose-5-phosphate, and interconvert 3–7 carbon sugars. The observation that *tald-1(RNAi)* induced the UPR^mt^ reporter and increased lifespan intrigued us, as transaldolase is not a mitochondrial protein and has not been previously implicated in longevity control in any organism. Here we report that transaldolase deficiency indeed alters mitochondrial function, as evidenced by changes in mitochondrial morphology and direct measurement of mitochondrial respiration. The lifespan extension from *tald-1(RNAi)* is independent of the UPR^mt^, and instead involves activation of an oxidative stress response mediated by the p38 MAPK PMK-1 and JNK MAPKs JNK-1 and KGB-1, and a concomitant starvation-like response that signals through the transcription factor EB (TFEB) homolog HLH-30. Furthermore, we find that activation of the starvation-like response transcriptionally activates HLH-30-dependent autophagy markers, increases autophagic flux, and increases expression of the longevity-promoting flavin-containing monooxygenase 2 (*fmo-2*).

## Results

### The pentose phosphate pathway modulates the UPR^mt^ and lifespan in *C*. *elegans*

**Fig 1:**
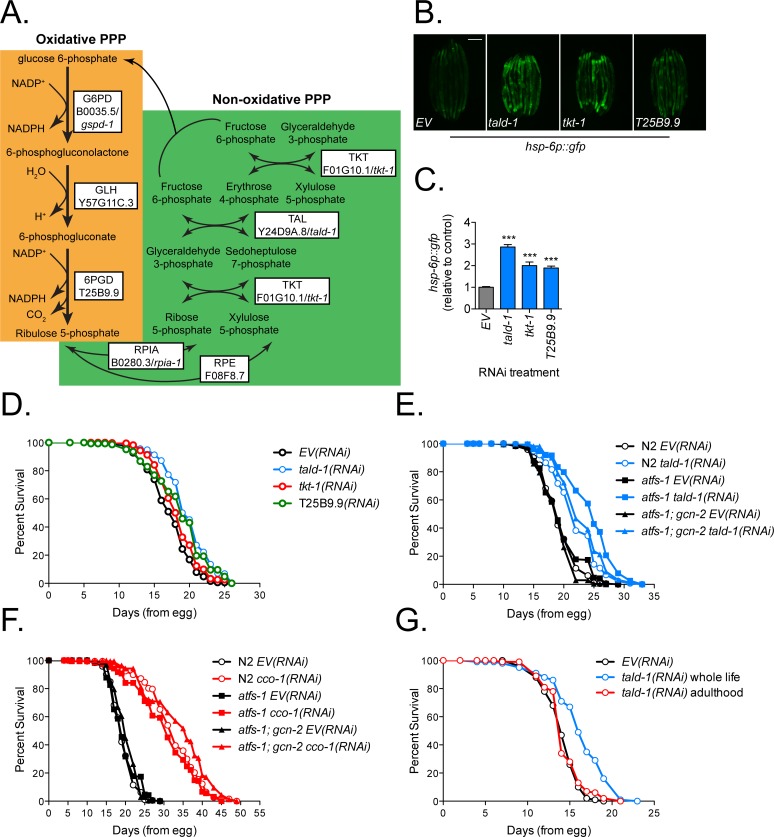
Inhibition of the pentose phosphate pathway activates the UPR^mt^ and extends lifespan. **(A)** Diagram of both the oxidative and non-oxidative branches of the PPP. The oxidative branch produces NADPH, while the non-oxidative branch produces ribose-5-P and interconverts sugar carbon backbones. The white boxes contain enzyme names with the human gene listed above the *C*. *elegans* homolog. **(B)** PPP gene knockdown increases *hsp-6p*::*gfp* reporter expression. **(C)** Mean relative fluorescence of *hsp-6p*::*gfp* animals grown on PPP RNAi. Fluorescence is calculated relative to *EV(RNAi)* controls (N = 4 independent experiments, pooled individual worm values, error bars indicate s.e.m., student’s t-test with Bonferroni’s correction). **(D)** RNAi knockdown of PPP genes extends *C*. *elegans* lifespan. N2 fed *EV(RNAi)* (mean 17.4±0.1 days, n = 455), N2 fed *tald-1(RNAi)* (mean 19.9±0.2 days, n = 391), N2 fed *tkt-1(RNAi)* (mean 18.4±0.1 days, n = 461), N2 fed T25B9.9*(RNAi)* (mean 18.8±0.2 days, n = 311). Lifespans were performed at 25°C, with pooled data from four independent experiments shown. **(E)** RNAi knockdown of *tald-1* extends lifespan independently of the UPR^mt^. N2 fed *EV(RNAi)* (mean 19.3±0.2 days, n = 192), N2 fed *tald-1(RNAi)* (mean 22.1±0.2 days, n = 251), *atfs-1(tm4525)* fed *EV(RNAi)* (mean 19.6±0.2 days, n = 230), *atfs-1(tm4525)* fed *tald-1(RNAi)* (mean 24.5±0.3 days, n = 228), *atfs-1(tm4525);gcn-2(ok871)* fed *EV(RNAi)* (mean 18.9±0.2 days, n = 205), *atfs-1(tm4525);gcn-2(ok871)* fed *tald-1(RNAi)* (mean 23.1±0.3 days, n = 220). Lifespans were performed at 20°C, with pooled data from two independent experiments shown. **(F)** RNAi knockdown of *cco-1* extends lifespan independently of the UPR^mt^. N2 fed *EV(RNAi)* (mean 19.3±0.2 days, n = 192), N2 fed *cco-1(RNAi)* (mean 32.3±0.5 days, n = 187), *atfs-1(tm4525)* fed *EV(RNAi)* (mean 19.6±0.2 days, n = 230), *atfs-1(tm4525)* fed *cco-1(RNAi)* (mean 29±0.6 days, n = 194), *atfs-1(tm4525);gcn-2(ok871)* fed *EV(RNAi)* (mean 18.9±0.2 days, n = 205), *atfs-1(tm4525);gcn-2(ok871)* fed *cco-1(RNAi)* (mean 32.6±0.5 days, n = 228). Lifespans were performed at 20°C, with pooled data from two independent experiments shown. **(G)** RNAi knockdown of *tald-1* extends lifespan only when knockdown occurs during development. N2 fed *EV(RNAi)* (mean 14.2±0.1 days, n = 361), N2 fed *tald-1(RNAi)* from hatching (mean 16.4±0.2 days, n = 468), N2 fed *tald-1(RNAi)* from L4 (mean 14.4±0.1 days, n = 330). Lifespans were performed at 25°C, with pooled data from three independent experiments shown. Lifespans in this figure are indicated as mean±s.e.m. and statistical analysis is provided in S1 Table. In this figure, statistics are displayed as: * *p*<0.05, ** *p*<0.01, *** *p*<0.001.

From an unbiased genome-wide RNAi screen for negative regulators of the mitochondrial unfolded protein response (UPR^mt^), we found that knockdown of either of the pentose phosphate pathway (PPP) enzymes transaldolase (*tald-1*) or transketolase (*tkt-1*) activates the UPR^mt^ reporter *hsp-6p*::*gfp* in *C*. *elegans* [15]. These enzymes function in the non-oxidative branch of the PPP, generating ribose-5-P for nucleotide synthesis and interconverting three, four, five, six, and seven carbon sugars (Fig 1A). To determine if *tald-1* and *tkt-1* deficiencies specifically cause mitochondrial stress independent of the PPP, we tested if knockdown of other PPP enzymes not detected in the initial RNAi screen could also induce the *hsp-6p*::*gfp* reporter. RNAi knockdown of T25B9.9, which encodes the oxidative PPP enzyme 6-phosphogluconate dehydrogenase (6PGD), caused a significant increase in *hsp-6p*::*gfp* expression (+89%), albeit less robustly than *tald-1(RNAi)* (+187%) (Fig 1B and 1C). In addition, RNAi knockdown of Y57G11C.3 (gluconolactone hydrolase/GLH) or *rpia-1* (ribose-5-phosphate isomerase/RPIA) slightly increased *hsp-6p*::*gfp* expression (+34%, +19%), while *gspd-1* (glucose-6-phosphate dehydrogenase/G6PD) RNAi did not (S1A Fig). Inhibition of the PPP at multiple enzymatic steps, both oxidative and non-oxidative, is therefore sufficient to increase expression of a mitochondrial stress reporter. Next, we asked if inhibition of the enzymatic steps that robustly activate *hsp-6p*::*gfp* increase lifespan similar to other RNAi clones that induce this reporter. We found that knockdown of *tald-1*, *tkt-1*, and T25B9.9/6PGD all increased lifespan (Fig 1D). Since *tald-1(RNAi)* resulted in the strongest phenotypes among PPP enzymes tested, we chose to focus our studies on understanding the mechanisms by which *tald-1* knockdown induces mitochondrial stress and enhances longevity.

The ATFS-1 transcription factor and the GCN-2 kinase, respectively, mediate the transcriptional and translational changes in response to mitochondrial stress that comprise the UPR^mt^ [11, 26]. Loss of either ATFS-1 or GCN-2 does not prevent the lifespan extension from mitochondrial inhibition [15, 16]. These factors act in a compensatory fashion, however, and GCN-2 may be able to establish mitochondrial protein homeostasis in the absence of ATFS-1 or vice versa. Therefore, to convincingly assess whether the UPR^mt^ regulates longevity from ETC or PPP inhibition, we examined if simultaneous loss of both *atfs-1* and *gcn-2* could prevent lifespan extension from RNAi knockdown of either *tald-1* or the complex IV subunit cytochrome c oxidase 1 gene, *cco-1*. Both RNAi clones significantly increased the lifespan of *atfs-1(tm4525); gcn-2(ok871)* animals comparable to their effects in wild-type nematodes (Fig 1E and 1F). Similar results were observed in *atfs-1(gk3094)* mutant animals (S1B and S1C Fig). Thus, we conclude that neither ATFS-1 nor GCN-2 are required for lifespan extension, further supporting the model that mitochondrial stress or ETC inhibition affect lifespan independently of the UPR^mt^.

We next examined the temporal and genetic requirements for *tald-1(RNAi)* lifespan extension in the context of previously described *C*. *elegans* longevity pathways. Like RNAi knockdown of ETC genes [4, 24], *tald-1(RNAi)* only extended lifespan when knockdown occurred during development (feeding beginning at L1), and adult-specific knockdown (feeding beginning at ~L4/young adult) had no effect on longevity (Fig 1G). Knockdown of *tald-1* also extended lifespan in animals carrying mutations of the FOXO-transcription factor *daf-16*, the AMP-activated protein kinase *aak-2*, and the germline-signaling factor *glp-1* (S2A–S2C Fig), consistent with the reported effects of mitochondrial RNAi treatments [4, 5, 8, 27, 28]. Interestingly, *tald-1(RNAi)* resulted in a larger lifespan extension in animals lacking the hypoxic response transcription factor HIF-1 (S2D Fig), while loss of *hif-1* attenuated the lifespan extension from *cco-1(RNAi)* (S2E Fig), as has been previously reported [20]. These data are consistent with a model that inhibition of the PPP extends lifespan by a mechanism that is overlapping but partially distinct from ETC inhibition.

### Transaldolase deficiency impairs mitochondrial respiration and increases oxidative stress *in vivo*

**Fig 2:**
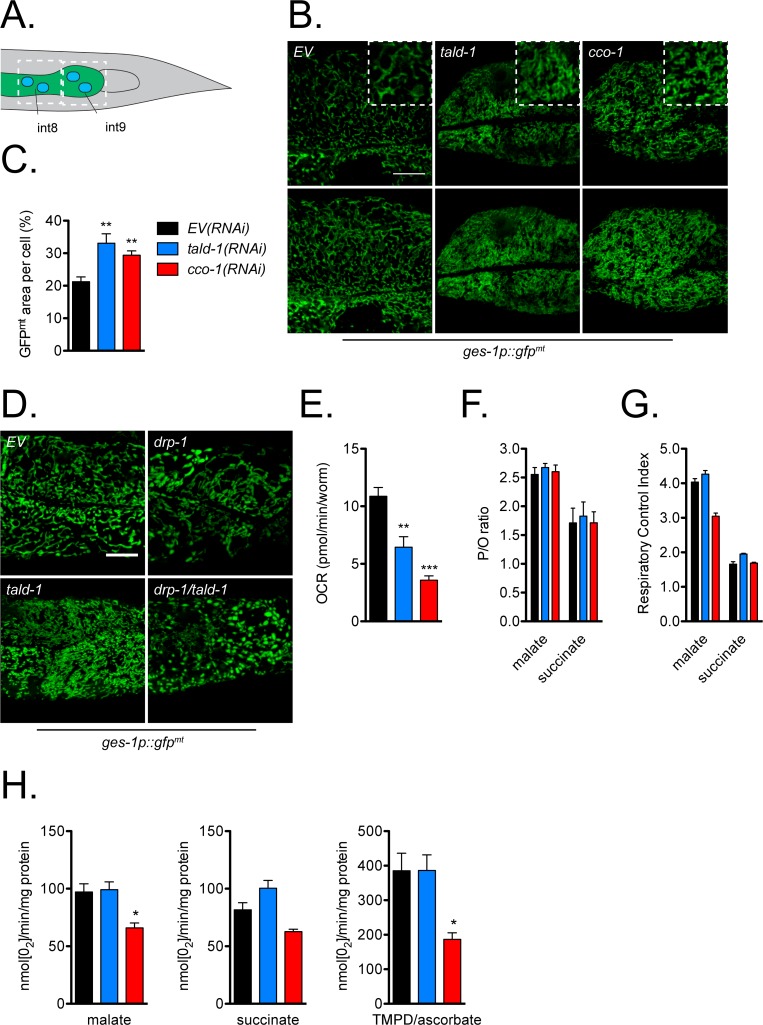
Transaldolase deficiency alters mitochondrial morphology and decreases *in vivo* mitochondrial respiration. **(A)** Diagram depicting the posterior intestinal cells that were visualized for mitochondrial morphology. **(B)** Intestinal mitochondrial morphology is altered by *tald-1(RNAi)* and *cco-1(RNAi)*. The top panel represents a single 0.34 μm slice imaged using confocal microscopy, with a magnified area displayed in a white dotted box to highlight morphology differences. The bottom panel consists of a max intensity projection of five z-slices to emphasize mitochondrial content in these cells. Scale bar, 10 μm. **(C)** Quantification of percent mitochondrial area per cell. (N = 2 independent experiments, error bars indicate s.e.m., student’s t-test with Bonferroni’s correction). **(D)** Mitochondrial morphology changes from *tald-1(RNAi)* are regulated by DRP-1. RNAi treatments include *EV(RNAi)*, *tald-1(RNAi)* [50:50 with *EV(RNAi)*], *drp-1(RNAi)* [50:50 with *EV(RNAi)*], and *tald-1(RNAi)* [50:50 with *drp-1(RNAi)*]. Scale bar, 10 μm. **(E)** Oxygen consumption rate decreases with *tald-1(RNAi)* and *cco-1(RNAi)*. OCR was measured using the Seahorse XF Analyzer and normalized to animal number (N = 6 independent experiments, error bars indicate s.e.m., student’s t-test with Bonferroni’s correction). **(F)** P/O ratio (the ATP produced per oxygen atom reduced), **(G)** respiratory control index (State 3:State 4 rates), **(H)** malate-driven respiration (Complex I-IV), succinate-driven respiration (Complex II-IV), and TMPD/ascorbate-driven respiration (Complex IV) were measured using the OXPHOS assay on isolated mitochondria from RNAi treated animals. Respiratory rates were measured as rate of disappearance of oxygen (nmol[O_2_]) per minute per mg protein (N = 4 independent experiments, error bars indicate s.e.m., student’s t-test with Bonferroni’s correction). Also, in this figure, color coating of bars and lines reflect the legend in (C).

Based on our findings that developmental knockdown of *tald-1* induced the UPR^mt^, we asked whether other parameters of mitochondrial function are affected by *tald-1(RNAi)*. First, we decided to use confocal microscopy to characterize any changes to intestinal mitochondrial morphology and content, since this tissue is particularly responsive to mitochondrial stress, as measured by the *hsp-6p*::*gfp* reporter. Using a mitochondrial-targeted GFP reporter whose expression is restricted to the intestine via the *ges-1* promoter, we observed that *tald-1(RNAi)* caused a disruption to normal mitochondrial morphology in intestinal cells (Fig 2A and 2B). Mitochondria in these animals became thin and smaller in size, reflecting a potential change in mitochondrial dynamics. A similar change in morphology occurred following *cco-1(RNAi)* (Fig 2B). Interestingly, despite the smaller size of mitochondria following *tald-1(RNAi)* and *cco-1(RNAi)*, there was increased GFP area per cell compared to controls (Fig 2C). This could indicate increased mitochondrial content; however, we did not observe any change in whole worm mitochondrial DNA abundance in these animals (S3A and S3B Fig), agreeing with previous studies reporting no change in mtDNA copy number from mitochondrial RNAi treatments [12, 29].

To better understand the effect of *tald-1(RNAi)* on mitochondrial morphology, we examined its interaction with factors known to regulate mitochondrial fusion and fission. As expected, knockdown of the fission factor dynamin-related GTPase *drp-1* (*DRP1*/*DNM1* homolog) caused intestinal mitochondria to swell and aggregate, while knockdown of the inner membrane fusion GTPase *eat-3* (*OPA1*/*MGM1* homolog) caused mitochondria to fragment and lack normal tubular structure (S4A Fig). Outer membrane fusion GTPase *fzo-1* (*MFN1*/*FZO1* homolog) knockdown also caused mitochondria to fragment, but morphology was remarkably similar to *tald-1(RNAi)* mitochondria, suggesting a mild pro-fission phenotype (S4A Fig). Accordingly, *drp-1(RNAi)* prevented the shift in mitochondrial morphology following *tald-1* knockdown (Fig 2D), indicating that the core fission machinery is required for this response. In contrast, *fzo-1* and the mitophagy components *pdr-1* (*PARK2* homolog) and *pink-1* (*PINK1* homolog) were not required for this phenotype (S4B and S4C Fig).

Since mitochondrial stress and mitochondrial fragmentation are associated with decreased mitochondrial function, we sought to directly measure metabolic activity in whole animals. The Seahorse XF24 Analyzer allows measurements of basal and real time changes in O_2_ consumption in *C*. *elegans* [30]. We found that knockdown of *tald-1* caused an approximately 41% reduction in oxygen consumption, while, as expected [8, 31], knockdown of *cco-1* caused a 67% reduction (Fig 2E). The reduction in oxygen consumption could not be fully explained by changes to worm length or density (S5A and S5B Fig), arguing that *tald-1(RNAi)* decreases basal mitochondrial respiration in whole animals.

To determine whether *tald-1(RNAi)* causes decreased mitochondrial respiration by altering ETC function or stability, mitochondria were isolated from animals and oxygen consumption of intact mitochondria was measured using malate, succinate, and TMPD/ascorbate as electron donors to drive complex I-, complex II-, and complex IV-dependent respirations, respectively. The mitochondria isolated in all trials retained normal coupling (P/O ratios) and a normal respiratory control index (State 3:State 4), indicating purification of healthy mitochondria (Fig 2F and 2G). As expected with Complex IV RNAi [32], *cco-1(RNAi)* decreased Complex I- and Complex IV-dependent respiration (Fig 2H). In contrast to *cco-1(RNAi)*, *tald-1(RNAi)* did not cause a change in any rates measured (Fig 2H). Therefore, *tald-1(RNAi)* decreases whole animal respiration without altering maximal ETC capacity, potentially by reducing equivalents to the ETC *in vivo*.

**Fig 3:**
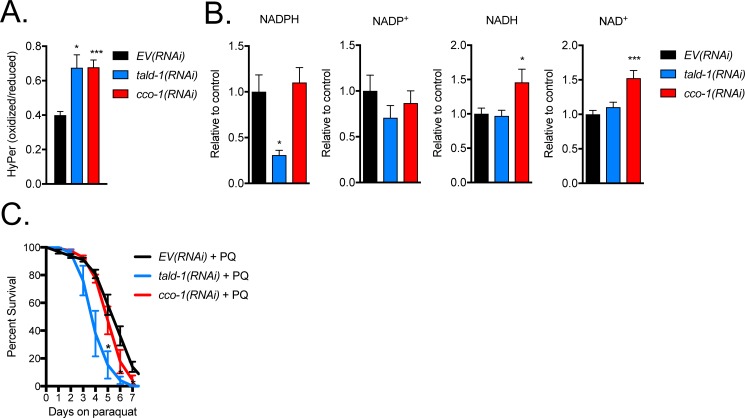
Redox stress is downstream of transaldolase deficiency. **(A)** H_2_O_2_ levels increase from RNAi knockdown of *tald-1* or *cco-1* (N = 7 independent experiments, error bars indicate s.e.m., student’s t-test with Bonferroni’s correction). **(B)** NADPH levels decrease from RNAi knockdown of *tald-1* (N = 5+ biological replicates, error bars indicate s.e.m., student’s t-test with Bonferroni’s correction). **(C)** RNAi knockdown of *tald-1* causes sensitivity to paraquat (PQ). Percent survival of N2 worms grown on RNAi bacteria and 10 mM PQ was measured over seven days. Survival analyses were performed at 25°C (N = 6 independent experiments, error bars indicate s.e.m., student’s t-test with Bonferroni’s correction). In this figure, statistics are displayed as: * *p*<0.05, ** *p*<0.01, *** *p*<0.001.

Mitochondrial dysfunction has been proposed to extend lifespan in *C*. *elegans* through increased production of ROS and altered redox signaling [20, 33–35]. To specifically observe *in vivo* changes in redox environment, we utilized a transgenic strain expressing the ratiometric H_2_O_2_-specific biosensor HyPer, which is comprised of the regulatory domain of the bacterial transcription factor OxyR (OxyR-RD) fused to circularly permuted yellow fluorescent protein [36]. The OxyR-RD of HyPer is selectively oxidized by H_2_O_2_, generating a disulphide bridge that consequently alters the fluorescent properties of cpYFP. RNAi knockdown of either *tald-1* or *cco-1* significantly increased the oxidation of HyPer as measured via a plate reader assay, indicating elevated cytoplasmic H_2_O_2_ levels in these animals (Fig 3A). By confocal microscopy we observed similar results for *tald-1(RNAi)*, but for *cco-1(RNAi)*, oxidation of the reporter did not reach statistical significance (p>0.1) (S6A and S6B Fig). Since the PPP generates cytosolic NADPH, we hypothesize that oxidative stress in *tald-1(RNAi)* animals results from NADPH depletion and reduced ROS buffering capacity. Using LC-MS to measure NAD metabolites (Table 1), we found that *tald-1(RNAi)* decreased cellular NADPH levels, whereas *cco-1(RNAi)* did not (Fig 3B). In accordance with higher endogenous levels of oxidative stress, *tald-1(RNAi)* animals were sensitive to 10mM paraquat treatment (a high dose on the hormetic curve of paraquat treatment that decreases wild-type lifespan [37, 38]), which leads to the production of mitochondrial superoxide (Fig 3C). Thus, the presence of a functional PPP is required for normal resistance to exogenous oxidative stress.

**Table 1:** Alkaline gradient (Run time 11 min).

Time (minutes)	Flow (ml/min)	%A	%B
0	0.5	5	95
6	0.5	39	61
8	0.5	56	44
8.2	0.5	73	27
9	0.5	5	95

### MAPKs mediate lifespan extension from transaldolase deficiency

**Fig 4:**
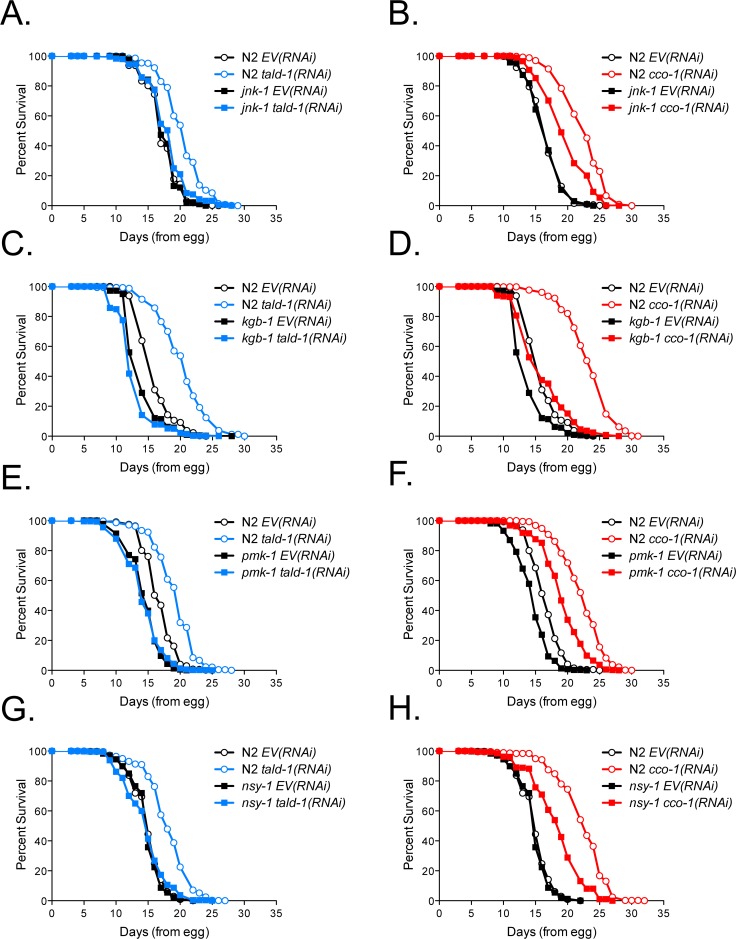
Lifespan extension from *tald-1(RNAi)* or *cco-1(RNAi)* requires stress-activated MAPKs. **(A)** RNAi knockdown of *tald-1* extends lifespan through the JNK MAPK JNK-1. N2 fed *EV(RNAi)* (mean 17.2±0.1 days, n = 506), N2 fed *tald-1(RNAi)* (mean 20.4±0.1 days, n = 500), *jnk-1(gk7)* fed *EV(RNAi)* (mean 17±0.1 days, n = 582), *jnk-1(gk7)* fed *tald-1(RNAi)* (mean 18.1±0.1 days, n = 488). Lifespans were performed at 25°C, with pooled data from five independent experiments shown. **(B)** RNAi knockdown of *cco-1* extends lifespan partially through the JNK MAPK JNK-1. N2 fed *EV(RNAi)* (mean 16.9±0.1 days, n = 494), N2 fed *cco-1(RNAi)* (mean 22.7±0.2 days, n = 431), *jnk-1(gk7)* fed *EV(RNAi)* (mean 16.1±0.1 days, n = 594), *jnk-1(gk7)* fed *cco-1(RNAi)* (mean 19.9±0.2 days, n = 408). Lifespans were performed at 25°C, with pooled data from four independent experiments shown. **(C)** RNAi knockdown of *tald-1* extends lifespan through the JNK MAPK KGB-1. N2 fed *EV(RNAi)* (mean 15±0.1 days, n = 630), N2 fed *tald-1(RNAi)* (mean 18.7±0.1 days, n = 657), *kgb-1(um3)* fed *EV(RNAi)* (mean 13.1±0.1 days, n = 580), *kgb-1* fed *tald-1(RNAi)* (mean 11.9±0.1 days, n = 600). Lifespans were performed at 25°C, with pooled data from four independent experiments shown. **(D)** RNAi knockdown of *cco-1* extends lifespan partially through the JNK MAPK KGB-1. N2 fed *EV(RNAi)* (mean 15±0.1 days, n = 630), N2 fed *cco-1(RNAi)* (mean 23.2±0.2 days, n = 511), *kgb-1(um3)* fed *EV(RNAi)* (mean 13.1±0.1 days, n = 580), *kgb-1* fed *cco-1(RNAi)* (mean 15.8±0.2 days, n = 501). Lifespans were performed at 25°C, with pooled data from four independent experiments shown. **(E)** RNAi knockdown of *tald-1* extends lifespan through the p38 MAPK PMK-1. N2 fed *EV(RNAi)* (mean 16.8±0.1 days, n = 494), N2 fed *tald-1(RNAi)* (mean 19.3±0.1 days, n = 460), *pmk-1(km25)* fed *EV(RNAi)* (mean 14.3±0.1 days, n = 514), *pmk-1(km25)* fed *tald-1(RNAi)* (mean 14±0.1 days, n = 525). Lifespans were performed at 25°C, with pooled data from four independent experiments shown. **(F)** RNAi knockdown of *cco-1* does not require the p38 MAPK PMK-1 for lifespan extension. N2 fed *EV(RNAi)* (mean 16±0.1 days, n = 575), N2 fed *cco-1(RNAi)* (mean 22.3±0.2 days, n = 448), *pmk-1(km25)* fed *EV(RNAi)* (mean 13.8±0.1 days, n = 609), *pmk-1(km25)* fed *cco-1(RNAi)* (mean 18.7±0.1 days, n = 535). Lifespans were performed at 25°C, with pooled data from four independent experiments shown. **(G)** RNAi knockdown of *tald-1* extends lifespan through the MAP3K NSY-1. N2 fed *EV(RNAi)* (mean 14.6±0.1 days, n = 542), N2 fed *tald-1(RNAi)* (mean 17.2±0.1 days, n = 599), *nsy-1(ag3)* fed *EV(RNAi)* (mean 14.9±0.1 days, n = 473), *nsy-1(ag3)* fed *tald-1(RNAi)* (mean 14.4±0.1 days, n = 508). Lifespans were performed at 25°C, with pooled data from four independent experiments shown. **(H)** RNAi knockdown of *cco-1* extends lifespan partially through the MAP3K NSY-1. N2 fed *EV(RNAi)* (mean 14.6±0.1 days, n = 542), N2 fed *cco-1(RNAi)* (mean 22.5±0.2 days, n = 454), *nsy-1(ag3)* fed *EV(RNAi)* (mean 14.9±0.1 days, n = 473), *nsy-1(ag3)* fed *cco-1(RNAi)* (mean 18.5±0.2 days, n = 458). Lifespans were performed at 25°C, with pooled data from four independent experiments shown. Lifespans in this figure are indicated as mean±s.e.m. and statistical analysis is provided in S1 Table.

Transaldolase deficiency in mammals causes a shift towards a more oxidative cellular redox status and compensatory activation of JNK MAPK signaling [39–41], prompting us to explore whether a similar response occurs in nematodes to regulate stress resistance and longevity. Remarkably, deletion of either *jnk-1* or *kgb-1*, which encode *C*. *elegans* JNK MAPKs, fully prevented the lifespan extension from *tald-1(RNAi)* and significantly attenuated the lifespan extension from *cco-1(RNAi)* (Fig 4A–4D). This effect was specific to mitochondrial longevity, since *daf-2(RNAi)* robustly extended the lifespan of *jnk-1(gk7)* and *kgb-1(um3)* animals (S7A and S7B Fig). Although deletion of either *jnk-1* or *kgb-1* prevented lifespan extension in response to either *tald-1(RNAi)* or *cco-1(RNAi)*, these mutations did not prevent the effects on mitochondrial respiration or UPR^mt^ induction (S7C–S7E Fig).

The p38 MAPK PMK-1 has been implicated in mitohormesis-induced lifespan extension in response to reduced insulin/IGF-1-like signaling, metformin treatment, or glycolysis inhibition [33, 35, 42]. Interestingly, PMK-1 was also required for lifespan extension from *tald-1(RNAi)*, but not from *cco-1(RNAi)* (Fig 4E and 4F). Therefore, despite some similar mitochondrial phenotypes and interactions with MAPK signaling, PPP inhibition and mitochondrial ETC RNAi longevity require both overlapping and distinct pathways. In addition, PMK-1 does not prevent UPR^mt^ induction from *tald-1(RNAi)* or *cco-1(RNAi)* (S8A and S8B Fig), suggesting it is not upstream of mitochondrial stress. As previously reported [42, 43], we also found that PMK-1 regulates *daf-2(RNAi)* lifespan extension (S8C Fig) and is not specific to PPP inhibition.

The MAP3K ASK1 is a well-established factor upstream of p38 and JNK MAPKs that responds to oxidative stress via interactions with redox proteins [44–46]. In *C*. *elegans*, the ASK1 homolog NSY-1 was found to act upstream of PMK-1, JNK-1, and KGB-1 in various contexts [47–52]. Accordingly, we found that loss of NSY-1 attenuated the lifespan extension from *tald-1(RNAi)* or *cco-1(RNAi)*, suggesting this factor responds to oxidative stress in both of these instances to promote longevity (Fig 4G and 4H). In agreement with NSY-1 regulating PMK-1 activity, we found that NSY-1 attenuated the lifespan extension from *daf-2(RNAi)* (S8D Fig). Therefore, NSY-1 is a MAP3K necessary for the activation of multiple longevity mechanisms, highlighting the importance of redox sensing in *C*. *elegans* longevity.

Since oxidative stress induces both p38 and JNK MAPK activity in mammalian cell lines, we predicted a similar response may occur in *C*. *elegans*. In order to test this, we treated animals with H_2_O_2_ and measured phosphorylation of JNK-1, KGB-1, and PMK-1 MAPKs. We found that as little as 5–15 minutes of H_2_O_2_ treatment is sufficient to activate these MAPKs (S8E Fig), demonstrating their high sensitivity to redox stress and further supporting for their role in longevity interventions associated with oxidative stress.

### Loss of transaldolase alters lipid metabolism and initiates a fasting-like response

**Fig 5:**
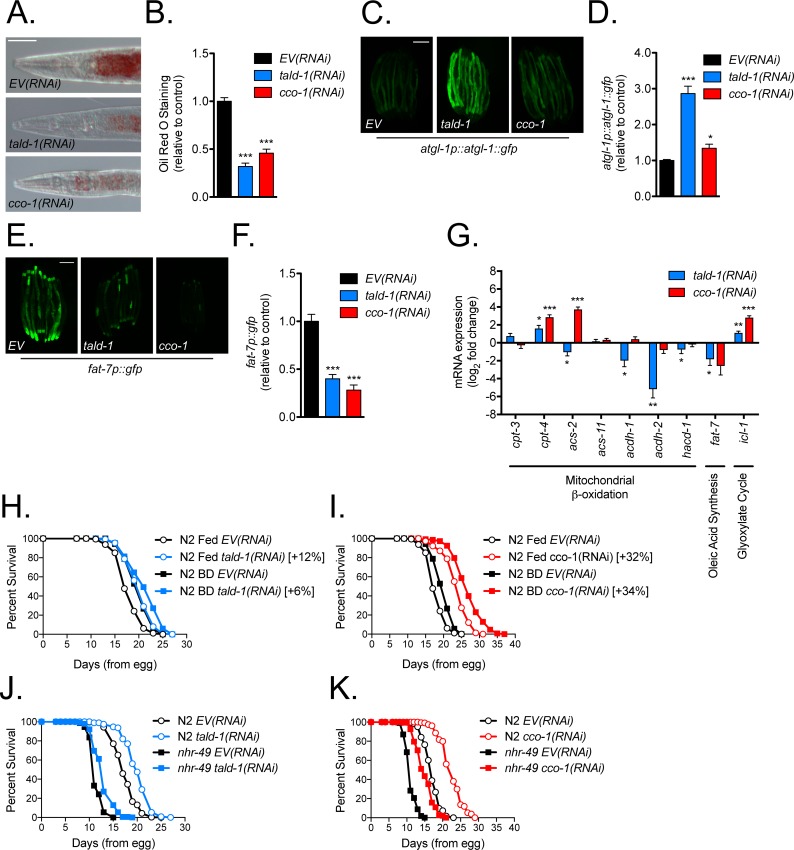
Transaldolase deficiency causes a starvation-like response that decreases animal fat content and rewires lipid metabolism gene expression. **(A)** Intestinal fat staining decreases from RNAi knockdown of *tald-1* or *cco-1*. Oil Red O (ORO) staining was performed on day 3 from hatching animals propagated at 20°C. Scale bar, 50 μm. **(B)** Quantification of ORO staining within anterior intestine (N = 2 independent experiments, pooled individual worm values, error bars indicate s.e.m., student’s t-test with Bonferroni’s correction). **(C)** RNAi knockdown of *tald-1* causes an increase in adipose triglyceride lipase ATGL-1 protein levels. Scale bar, 200 μm. **(D)** Mean relative fluorescence of ATGL-1::GFP signal in animals grown on *tald-1(RNAi)* or *cco-1(RNAi)*. Fluorescence is calculated relative to *EV(RNAi)* controls (N = 4 independent experiments, pooled individual worm values, error bars indicate s.e.m., student’s t-test with Bonferroni’s correction). **(E)** RNAi knockdown of *tald-1* or *cco-1* causes a decrease in stearoyl-CoA desaturase *fat-7p*::*gfp* reporter expression. Scale bar, 200 μm. **(F)** Mean relative fluorescence of *fat-7p*::*gfp* reporter animals grown on *tald-1(RNAi)* or *cco-1(RNAi)*. Fluorescence is calculated relative to *EV(RNAi)* controls (N = 3 independent experiments, pooled individual worm values, error bars indicate s.e.m., student’s t-test with Bonferroni’s correction). **(G)** Gene expression of starvation-responsive lipid metabolism genes is altered in *tald-1(RNAi)* animals. Log2 fold change calculated to emphasize the increases and decreases in gene expression levels from RNAi treatments (N = 6–8 independent experiments, error bars indicate s.e.m., paired student’s t-tests with Bonferroni’s correction). **(H)** RNAi knockdown of *tald-1* does not robustly extend lifespan of BD animals. N2 fed *EV(RNAi)* (mean 18.2±0.2 days, n = 161), N2 fed *tald-1(RNAi)* (mean 20.4±0.2 days, n = 151), BD animals developed on *EV(RNAi)* (mean 20.2±0.2 days, n = 123), BD animals developed on *tald-1(RNAi)* (mean 21.5±0.3 days, n = 150). Lifespans were performed at 25°C, with one experiment shown. **(I)** RNAi knockdown of *cco-1* extends lifespan dissimilar from BD. N2 fed *EV(RNAi)* (mean 18.2±0.2 days, n = 161), N2 fed *cco-1(RNAi)* (mean 24±0.3 days, n = 156), BD animals developed on *EV(RNAi)* (mean 20.2±0.2 days, n = 123), BD animals developed on *cco-1(RNAi)* (mean 27.1±0.3 days, n = 148). Lifespans were performed at 25°C, with one experiment shown. **(J)** RNAi knockdown of *tald-1* does not require NHR-49 for lifespan extension. N2 fed *EV(RNAi)* (mean 17.5±0.1 days, n = 366), N2 fed *tald-1(RNAi)* (mean 20.1±0.1 days, n = 397), *nhr-49(nr2041)* fed *EV(RNAi)* (mean 11.5±0.1 days, n = 310), *nhr-49(nr2041)* fed *tald-1(RNAi)* (mean 12.9±0.1 days, n = 333). Lifespans were performed at 25°C, with pooled data from three independent experiments shown. **(K)** RNAi knockdown of *cco-1* does not require NHR-49 for lifespan extension. N2 fed *EV(RNAi)* (mean 17±0.1 days, n = 532), N2 fed *cco-1(RNAi)* (mean 22.6±0.2 days, n = 344), *nhr-49(nr2041)* fed *EV(RNAi)* (mean 11.1±0.1 days, n = 495), *nhr-49(nr2041)* fed *cco-1(RNAi)* (mean 15±0.1 days, n = 489). Lifespans were performed at 25°C, with pooled data from four independent experiments shown. Lifespans in this figure are indicated as mean±s.e.m. and statistical analysis is provided in S1 Table. In this figure, statistics are displayed as: * *p*<0.05, ** *p*<0.01, *** *p*<0.001.

In addition to reducing *in vivo* respiration rates, we noted that *tald-1(RNAi)* and *cco-1(RNAi)* also caused dramatic reductions in intestinal fat levels, as assessed by Oil Red O (ORO) staining (Fig 5A and 5B). Such a response could reflect decreased lipid synthesis, increased fatty acid oxidation (associated with starvation), or decreased fatty acid absorption. Because *C*. *elegans* acquire the majority of lipid species from their bacterial diet and not from *de novo* fatty acid synthesis, with the exception of monomethyl branched-chain fatty acids [53], we focused on determining whether there were changes in expression of metabolic genes regulated by starvation including lipases, β-oxidation, monounsaturated fatty acid synthesis, and glyoxylate pathway genes [54].

First, we examined if decreased ORO staining might reflect degradation of cytoplasmic lipid droplets. The adipose triglyceride lipase ATGL-1 is an important lipase that is stabilized and localized to lipid droplets during fasting to mediate lipolysis [55]. Using the *atgl-1p*::*atgl-1*::*gfp* translational reporter, we found that *tald-1(RNAi)* dramatically increased ATGL-1::GFP levels, suggesting enhanced breakdown of lipid droplets in these animals (Fig 5C and 5D). The stearoyl-CoA desaturase *fat-7* controls the relative abundance of saturated and mono-unsaturated fatty acids by converting stearic acid (18:0) to oleic acid (18:1). Expression of *fat-7* is positively regulated by NHR-49 in fed conditions but is repressed during starvation, independent of NHR-49, to preserve saturated fatty acid levels [54, 56]. Using the *fat-7p*::*gfp* reporter we found that *fat-7* expression was dramatically repressed in *tald-1(RNAi)* or *cco-1(RNAi)* animals (Fig 5E and 5F). This observation was also confirmed by qRT-PCR (Fig 5G). In a similar fashion, other metabolic genes known to be regulated by starvation [47, 54], such as genes involved in β-oxidation and the glyoxylate pathway, also change in *tald-1(RNAi)* animals and *cco-1(RNAi)* animals (Fig 5G). For example, we observed increased expression of carnitine palmitoyltransferase 4 (*cpt-4*) following *tald-1(RNAi)* or *cco-1(RNAi)*, suggesting increased import of long-chain fatty acids into the mitochondria (Fig 5G). In addition, we observed increased expression of the bifunctional glyoxylate gene *icl-1* with *tald-1(RNAi)* or *cco-1(RNAi)*, indicating increased metabolism of fatty acids to promote gluconeogenesis and generation of succinate without concomitant NAD^+^ consumption and carbon loss (Fig 5G). In some cases, directionality or robustness of gene expression differed between *tald-1(RNAi)* and *cco-1(RNAi)* animals. For example, *acs-2* expression is decreased by *tald-1(RNAi)* and increased by *cco-1(RNAi)*, while *acdh-1*, *acdh-2*, and *hacd-1* are downregulated by *tald-1(RNAi)*, but not *cco-1(RNAi)* (Fig 5G). Multiple genes exist for each enzyme involved in β-oxidation in *C*. *elegans* and depending on the type of starvation response differential regulation of isoforms and even downregulation of certain β-oxidation genes occurs possibly owing to tissue-specific alterations or isoform preference for certain fatty acid chain lengths [47, 54, 57–59]. Thus, it is not surprising that metabolic gene expression profiles in *tald-1(RNAi)* and *cco-1(RNAi)* animals differ in some regards.

**Fig 6:**
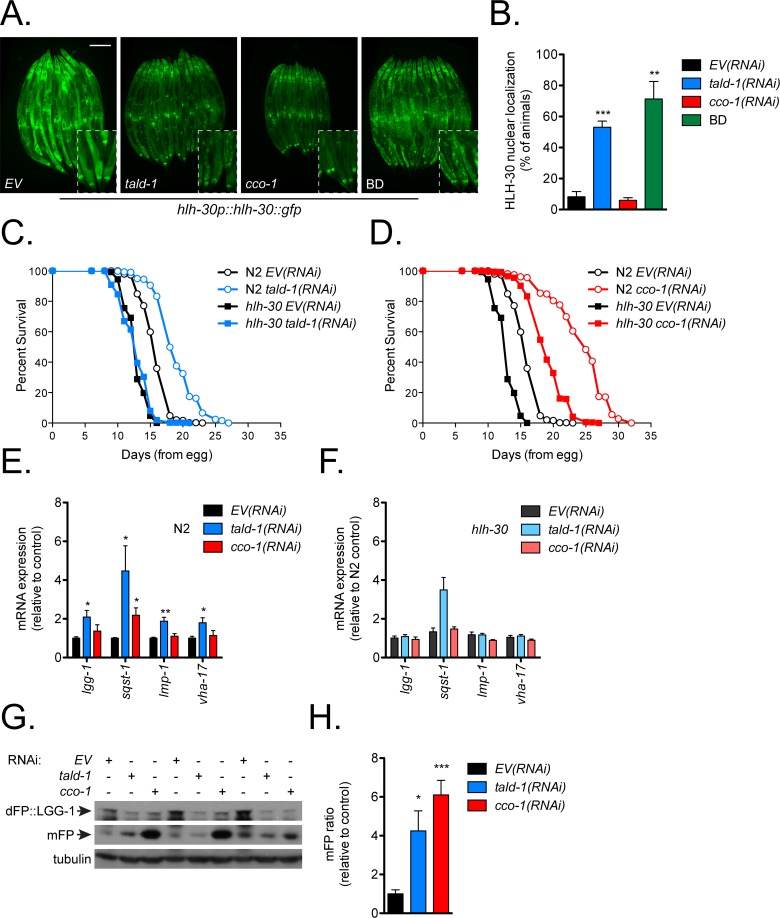
HLH-30 mediates the lifespan extension and autophagy gene expression from *tald-1(RNAi)*. **(A)** RNAi knockdown of *tald-1* increases nuclear localization of HLH-30 similarly to starvation. BD animals were starved for 8 hours on FUDR plates prior to imaging. Scale bar, 200 μm. **(B)** Percent of animals displaying HLH-30 nuclear localization. (N = 8 independent experiments, error bars indicate s.e.m., student’s t-test with Bonferroni’s correction). **(C)** HLH-30 is required for the lifespan extension from *tald-1(RNAi)*. N2 fed *EV(RNAi)* (mean 16±0.1 days, n = 476), N2 fed *tald-1(RNAi)* (mean 19.2±0.1 days, n = 455), *hlh-30(tm1978)* fed *EV(RNAi)* (mean 12.9±0.1 days, n = 510), *hlh-30(tm1978)* fed *tald-1(RNAi)* (mean 12.9±0.1 days, n = 514). Lifespans were performed at 25°C, with pooled data from four independent experiments shown. **(D)** HLH-30 is not required for the lifespan extension from *cco-1(RNAi)*. N2 fed *EV(RNAi)* (mean 16±0.1 days, n = 476), N2 fed *cco-1(RNAi)* (mean 24.3±0.2 days, n = 362), *hlh-30(tm1978)* fed *EV(RNAi)* (mean 12.9±0.1 days, n = 510), *hlh-30(tm1978)* fed *cco-1(RNAi)* (mean 19.2±0.1 days, n = 533). Lifespans were performed at 25°C, with pooled data from four independent experiments shown. **(E)** Gene expression of autophagy genes is upregulated in *tald-1(RNAi)* animals (N = 6 biological replicates, error bars indicate s.e.m., student’s t-test with Bonferroni’s correction). **(F)** HLH-30 is required for the upregulation of autophagy genes by *tald-1(RNAi)*. qRT-PCR was performed on RNA isolated from *hlh-30(tm1978)* animals (N = 3 biological replicates, error bars indicate s.e.m., student’s t-test with Bonferroni’s correction). **(G)** Autophagic flux increases from RNAi knockdown of *tald-1* or *cco-1*. Western blot analysis was performed on protein lysates from *eft-3p*::*dFP*::*lgg-1* animals using an anti-GFP antibody to detect full-length dFP-LGG-1 and monomeric FP. An anti-α-tubulin antibody was used as a loading control. Three biological replicates for each RNAi treatment are presented. **(H)** Quantification of the dFP::LGG-1 ratiometric reporter. The intensity of monomeric FP to full-length dFP::LGG-1 was measured to determine autophagic flux (N = 5 independent experiments, error bars indicate s.e.m., student’s t-test with Bonferroni’s correction). Lifespans in this figure are indicated as mean±s.e.m. and statistical analysis is provided in S1 Table. In this figure, statistics are displayed as: * *p*<0.05, ** *p*<0.01, *** *p*<0.001.

To explore if the starvation-like metabolic response underlies the pro-longevity effects of *tald-1(RNAi)* or *cco-1(RNAi)*, we performed epistasis analyses with dietary restricted animals and starvation responsive transcription factors NHR-49 and HLH-30. We found that *tald-1(RNAi)* lifespan extension is slightly additive (mean +6% extension, median +0%) to complete removal of the bacterial food source in adulthood (bacterial deprivation, BD), suggesting that *tald-1(RNAi)* functions through a starvation response (Fig 5H). Supporting the notion that mitochondrial RNAi functions independently of dietary restriction to extend lifespan (mitochondrial RNAi acts during development [4, 24], whereas BD acts during adulthood [60, 61]), we found that *cco-1(RNAi)* was fully additive to BD lifespan extension (Fig 5I). This is intriguing since we observed that *tald-1(RNAi)* only extends lifespan when RNAi is initiated from development similar to mitochondrial RNAi. One possibility is that TALD-1 protein levels must reach a lower threshold to ensure hormetic benefits and a starvation response during adulthood, which is more likely if RNAi treatment begins from hatching. NHR-49 is a master regulator of gene expression changes that enable the mobilization of fat for energy metabolism, and HLH-30 regulates autophagy, fat storage, and has been previously implicated in lifespan extension downstream of dietary restriction and insulin/IGF-1-like signaling [62–64]. Interestingly, NHR-49 is not required for the lifespan effect of either *tald-1(RNAi)* or *cco-1(RNAi)* (Fig 5J and 5K). This agrees with a previous study that found reduced complex I, III, and IV activity caused NHR-49 dependent gene expression changes and increased lifespan independent of NHR-49 [65]. In contrast, *tald-1(RNAi)* caused nuclear localization of HLH-30 similar to starvation (Fig 6A and 6B), and also required HLH-30 for lifespan extension (Fig 6C). Importantly, *tald-1(RNAi)* did not affect food consumption, as measured by pharyngeal pumping rate (S9A Fig). In contrast to *tald-1(RNAi)*, *cco-1(RNAi*) did not induce HLH-30 nuclear localization and the lifespan extension in this case was independent of HLH-30 (Fig 6A, 6B and 6D). Thus, transaldolase deficiency induces a starvation-like response and requires the autophagy regulator TFEB/HLH-30 for lifespan extension.

### HLH-30 activates autophagy and flavin-containing monooxygenase 2 in response to transaldolase deficiency

One major function of TFEB/HLH-30 is to promote autophagy [62, 63, 66], and this activity of HLH-30 is necessary for lifespan extension in response to dietary restriction and reduced insulin/IGF-1-like signaling [62]. Consistent with our observation that *tald-1(RNAi)* induces nuclear localization of HLH-30, we found that components of the autophagy pathway [62] were upregulated in a HLH-30-dependent fashion, including *lgg-1* (*LC3* homolog), *sqst-1* (p62/*SQSTM1* homolog), *lmp-1* (*LAMP1* homolog), and lysosomal subunit *vha-17* (Fig 6E and 6F). In addition, autophagic flux is increased by *tald-1(RNAi)* (Fig 6G and 6H), as measured by a recently described LGG-1 reporter of lysosomal protease activity [67]. The reporter consists of LGG-1 tagged with two fluorescent proteins containing a flexible protease-sensitive linker. When the lysosome fuses with the autophagosome, lysosomal proteases cleave dFP::LGG-1 and release protease-resistant monomeric FP (mFP). Increases in autophagic flux are thereby reflected as an increase in the [mFP]/[dFP::LGG-1] ratio.

**Fig 7:**
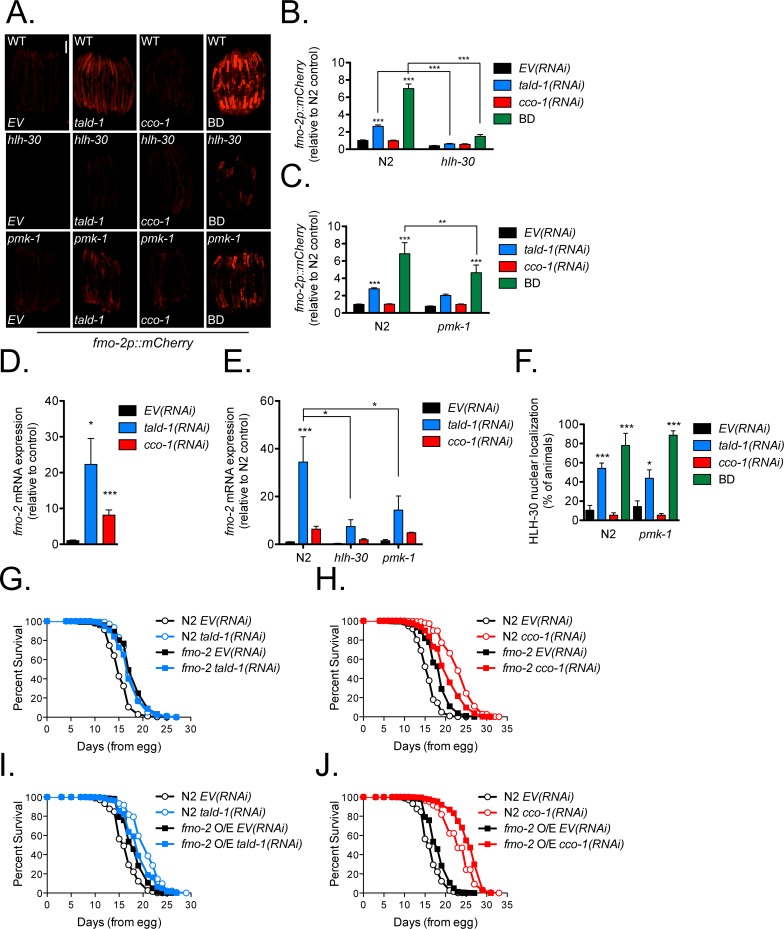
The flavin-containing monooxygenase FMO-2 is upregulated in a HLH-30 and PMK-1 dependent fashion and regulates the lifespan extension from *tald-1(RNAi)*. **(A)** *fmo-2p*::*mCherry* reporter expression is increased by *tald-1(RNAi)* or BD in a HLH-30 and PMK-1 dependent fashion. BD animals were starved for 24 hours on FUDR plates prior to imaging. Scale bar, 200 μm. **(B)** Mean relative fluorescence of *fmo-2p*::*mCherry* reporter animals in the context of the *hlh-30(tm1978)* mutation. Fluorescence is calculated relative to N2 *EV(RNAi)* controls (N = 3 independent experiments, pooled individual worm values, error bars indicate s.e.m., ANOVA with Bonferroni’s post-hoc). **(C)** Mean relative fluorescence of *fmo-2p*::*mCherry* reporter animals in the context of the *pmk-1(km25)* mutation. Fluorescence is calculated relative to N2 *EV(RNAi)* controls (N = 5 independent experiments, pooled individual worm values, error bars indicate s.e.m., ANOVA with Bonferroni’s post-hoc). **(D)** Gene expression of *fmo-2* is upregulated by *tald-1(RNAi)* or *cco-1(RNAi)* (N = 11 biological replicates, error bars indicate s.e.m., student’s t-test with Bonferroni’s correction). **(E)** Gene expression of *fmo-2* is upregulated by *tald-1(RNAi)* in a HLH-30 and PMK-1 dependent fashion (N = 3–6 biological replicates, error bars indicate s.e.m., ANOVA with Bonferroni’s post-hoc). **(F)** Percent of animals displaying HLH-30 nuclear localization. BD animals were starved for 8 hours on FUDR plates prior to imaging (N = 5 independent experiments, error bars indicate s.e.m., ANOVA with Bonferroni’s post-hoc). **(G)** FMO-2 is required for the lifespan extension from *tald-1(RNAi)*. N2 fed *EV(RNAi)* (mean 15.3±0.1 days, n = 341), N2 fed *tald-1(RNAi)* (mean 17.8±0.1 days, n = 353), *fmo-2(ok2147)* fed *EV(RNAi)* (mean 18±0.2 days, n = 314), *fmo-2(ok2147)* fed *tald-1(RNAi)* (mean 17.4±0.2 days, n = 382). Lifespans were performed at 25°C, with pooled data from three independent experiments shown. **(H)** FMO-2 is partially required for the lifespan extension from *cco-1(RNAi)*. N2 fed *EV(RNAi)* (mean 15.7±0.1 days, n = 562), N2 fed *cco-1(RNAi)* (mean 23.3±0.2 days, n = 616), *fmo-2(ok2147)* fed *EV(RNAi)* (mean 18.3±0.1 days, n = 474), *fmo-2(ok2147)* fed *cco-1(RNAi)* (mean 20.5±0.2 days, n = 473). Lifespans were performed at 25°C, with pooled data from five independent experiments shown. **(I)** Lifespan extension from *fmo-2* overexpression is not additive with *tald-1(RNAi)*. N2 fed *EV(RNAi)* (mean 16.5±0.1 days, n = 453), N2 fed *tald-1(RNAi)* (mean 20.6±0.1 days, n = 421), *eft-3p*::*fmo-2* fed *EV(RNAi)* (mean 18.2±0.1 days, n = 439), *eft-3p*::*fmo-2* fed *tald-1(RNAi)* (mean 19.1±0.1 days, n = 435). Lifespans were performed at 25°C, with pooled data from three independent experiments shown. **(J)** Lifespan extension from *fmo-2* overexpression is additive with *cco-1(RNAi)*. N2 fed *EV(RNAi)* (mean 16.5±0.1 days, n = 453), N2 fed *cco-1(RNAi)* (mean 23.3±0.2 days, n = 259), *eft-3p*::*fmo-2* fed *EV(RNAi)* (mean 18.2±0.1 days, n = 439), *eft-3p*::*fmo-2* fed *cco-1(RNAi)* (mean 25.5±0.2 days, n = 352). Lifespans were performed at 25°C, with pooled data from three independent experiments shown. Lifespans in this figure are indicated as mean±s.e.m. and statistical analysis is provided in S1 Table. In this figure, statistics are displayed as: * *p*<0.05, ** *p*<0.01, *** *p*<0.001.

Another important target of HLH-30 recently implicated in longevity control is the flavin-containing monooxygenase FMO-2. FMO-2 is induced by both hypoxic signaling and starvation, and its induction by starvation is dependent on HLH-30 [68]. Utilizing an *fmo-2p*::*mCherry* transcriptional reporter, we found that *tald-1(RNAi)* also robustly induced *fmo-2* expression, although not to the same extent as complete removal of the bacterial food source (Fig 7A–7C). Unexpectedly, whereas, *tald-1(RNAi)* or starvation causes intestinal *fmo-2* expression, *cco-1(RNAi)* causes *fmo-2* expression in the pharynx and cells proximal to the anterior bulb (S10A Fig). The increased expression of *fmo-2* indicated by the reporter was confirmed by qRT-PCR (Fig 7D).

Since the regulation of *fmo-2* is not well understood, we decided to test if HLH-30 is an essential regulatory factor of *fmo-2* in multiple contexts. In support of this, we found that HLH-30 mediates *fmo-2* expression from both BD and *tald-1(RNAi)* (Fig 7A and 7B). This observation was supported by qRT-PCR (Fig 7E). Thus, we decided to use the *fmo-2* transcriptional reporter as a proxy for HLH-30 activity to determine genetic relationships between HLH-30 and the MAPKs that mediate *tald-1(RNAi)* lifespan extension. JNK-1 and KGB-1 were not required for *fmo-2p*::*mCherry* induction from *tald-1(RNAi)* or BD (S10B Fig), arguing that these MAPKs are not upstream of HLH-30. However, the p38 MAPK PMK-1 was required for induction of *fmo-2p*::*mCherry* from BD and there was a similar trend for *tald-1(RNAi)* (Fig 7A and 7C). Supporting this, *fmo-2* induction by *tald-1(RNAi)* was attenuated in *pmk-1(km25)* animals by qRT-PCR (Fig 7E). Since loss of function in *hlh-30* and *pmk-1* cause similar effects with respect to *fmo-2* expression and lifespan epistasis with *tald-1(RNAi)* and *cco-1(RNAi)*, we tested if PMK-1 is upstream of HLH-30. Surprisingly, we found that *pmk-1(km25)* mutation did not alter HLH-30 nuclear localization from *tald-1(RNAi)* or BD (Fig 7F). Therefore, the simplest model is that PMK-1 functions in parallel with HLH-30 to activate *fmo-2* expression.

To determine if FMO-2 activation contributes to the lifespan extension from transaldolase deficiency, we treated *fmo-2(ok2147)* mutants with *tald-1(RNAi)*. We found that *tald-1(RNAi)* did not extend the lifespan of *fmo-2(ok2147)* animals (Fig 7G). In addition, *cco-1(RNAi)* longevity partially required *fmo-2* (Fig 7H). In neither case did deletion of *fmo-2* affect induction of the UPR^mt^ reporter (S10C Fig). Consistent with the model that FMO-2 acts downstream of *tald-1(RNAi)* to promote longevity, *tald-1(RNAi)* did not further extend the lifespan of long-lived *eft-3p*::*fmo-2* animals ubiquitously overexpressing *fmo-2* (Fig 7I).

## Discussion

**Fig 8:**
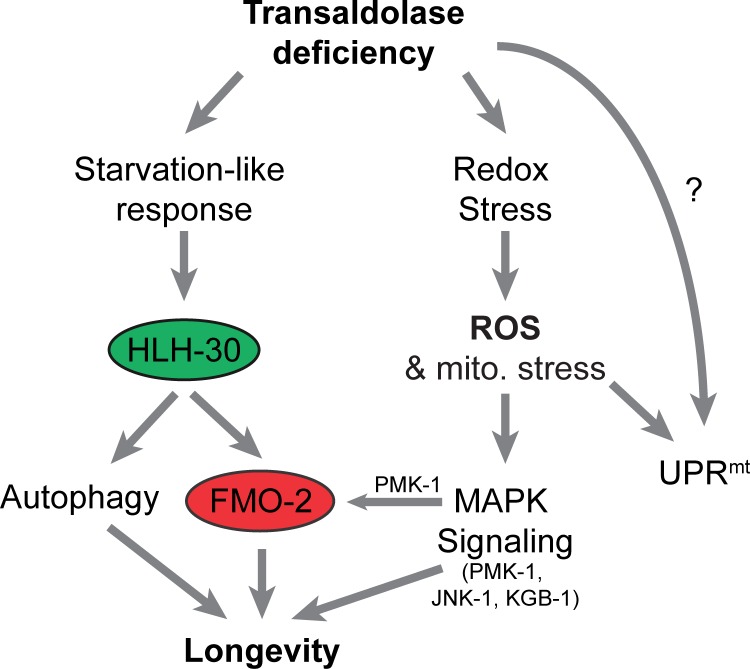
Model of transaldolase deficiency mediated longevity. Reduced activity of the pentose phosphate pathway enzyme transaldolase has several consequences, including inhibition of mitochondrial respiration, induction of a mitochondrial stress response, alterations in redox homeostasis, and activation of a starvation-like metabolic response. Lifespan extension in response to transaldolase deficiency appears to be mediated by both MAPK signaling and HLH-30 mediated induction of autophagy and activation of FMO-2.

In this study, we found that the inhibition of the PPP enzyme transaldolase impairs mitochondrial respiration, induces a starvation-like metabolic response, and activates MAPK signaling pathways that together promote longevity in *C*. *elegans*. These observations define unexpected new connections between the cytosolic PPP, mitochondrial metabolism, and aging. Although our interest in transaldolase stemmed from the observation that *tald-1(RNAi)* induces the UPR^mt^, activation of this mitochondrial stress response does not appear to be involved in mediating the longevity phenotype. Instead, lifespan extension from *tald-1(RNAi)* likely involves at least two outputs previously associated with longevity: induction of autophagy and activation of the flavin-containing monooxygenase 2 (Fig 8).

The relationship between PPP activity and mitochondrial function is particularly intriguing. Our studies indicate that inhibition of the PPP is sufficient to reduce respiration rates *in vivo* and remodel the mitochondrial network by activating mitochondrial fission, but importantly, this is accomplished without apparent functional changes to the ETC itself, as evidenced by the normal *in vitro* activity of purified mitochondria. This mechanistically differentiates *tald-1(RNAi)* from the well-characterized long-lived ETC-deficient animals such as *cco-1(RNAi)* and *isp-1(qm150)*, which directly impair ETC structure and function [32, 69]. Our findings also support mammalian literature where mitochondrial function is altered by transaldolase deficiency. For example, lymphoblasts isolated from transaldolase deficient patients exhibit decreased mitochondrial membrane potential, increased mitochondrial mass, and increased H_2_O_2_ levels, while transaldolase deficient mice are infertile due to mitochondrial defects in spermatozoa [39, 40]. Although the UPR^mt^ is apparently not involved in mediating the lifespan effects, its activation clearly indicates mitochondrial stress *in vivo* in the *tald-1(RNAi)* animals. One potential source of this mitochondrial stress could be increased levels of ROS, as indicated by the HyPer reporter and the enhanced sensitivity of *tald-1(RNAi)* animals to paraquat.

These findings highlight the importance of the PPP not only as a key pathway involved in central carbon metabolism, but also as a signaling hub. This close monitoring of PPP activity is logical, as it lies at the intersection of nucleotide metabolism, fatty acid/sterol synthesis, redox regulation, and glycolysis. In this light, the starvation like-response to *tald-1(RNAi)* is of particular interest, since it suggests that decreased PPP flux is monitored by the cell and results in diminished growth signaling. We speculate this occurs at least partially through decreased mTORC1 signaling, as we observed increased autophagic flux and activation of HLH-30, which is negatively regulated by mTORC1 [63, 70–73]. Furthermore, this starvation-like response caused a metabolic shift that depleted intestinal fat stores and rewired lipid metabolism to downregulate the stearoyl-CoA desaturase (Δ-9-desaturase, SCD) *fat-7*, upregulate mitochondrial fatty acid import genes, and the glyoxylate gene *icl-1*, among others. A reduction in *fat-7* expression limits monounsaturated fatty acid synthesis, which maintains saturated fatty acid levels, but could also alter cellular and membrane lipid composition, including that of the mitochondria [74]. Alternatively, decreased *fat-7* levels may indicate one arm of a concerted effort to breakdown fats through gene expression changes, as *fat-7* negatively regulates β-oxidation [56, 58]. We suspect this gene expression program promotes the mobilization and breakdown of fatty acids for both energy metabolism and gluconeogenesis through the mitochondrial glyoxylate pathway [75].

In this study, we implicated stress-activated MAPKs as one class of sensors that respond to reduced PPP activity and appear to be independent of HLH-30 activity. It is unclear whether direct interactions between enzymes or products of the PPP regulate MAPKs or if multiple indirect steps connect their activities. NADPH produced by the PPP not only maintains a reduced cytosolic redox environment, but also affects antioxidant systems such as thioredoxin, glutaredoxin, and peroxiredoxin that respond to oxidative stress via thiol-based chemistry to initiate downstream signaling events. For example, activity of the MAP3K ASK1/NSY-1 is fine-tuned via thiol-disulphide exchange reactions mediated by these redox proteins [76–80]. Thus, we speculate that a shift to a more oxidative cytosolic redox from PPP inhibition is coupled to activation of ASK1/NSY-1 and downstream p38 and JNK MAPK signaling. Accordingly, in a context dependent fashion, *C*. *elegans* p38 and JNK MAPKs regulate stress resistance from various oxidative insults and longevity from dietary restriction interventions such as intermittent fasting and metformin treatment [35, 47]. Our data further confirms that elevated cytosolic H_2_O_2_ correlates with MAPK mediated lifespan extension in novel and distinct contexts: RNAi knockdown of a PPP enzyme and an ETC Complex IV subunit. Interestingly, the MAP3K NSY-1 was required for the full lifespan extension from both interventions, but differences existed for downstream MAPK requirements. For example, *tald-1(RNAi)* required both the p38 MAPK PMK-1 and the JNK MAPKs JNK-1 and KGB-1 for lifespan extension, while *cco-1(RNAi)* only required the JNK MAPK branch. Furthermore, our discovery of an unreported role for the JNK MAPK pathway in mediating ETC RNAi longevity is intriguing, as no other genes outside *hif-1* and the p38 MAPK *pmk-3* have been reported to mediate these effects in *C*. *elegans* [20].

The simplest model for enhanced longevity downstream of *tald-1(RNAi)* is through activation of HLH-30, which has been previously shown to promote longevity downstream of dietary restriction, mTOR signaling, and insulin/IGF-1-like signaling [62]. Prior studies have focused primarily on activation of autophagy and lipophagy by HLH-30 [62, 63], but we recently reported that FMO-2 is another important pro-longevity HLH-30 target that is activated by both dietary restriction and the hypoxic response [68]. The exact role of FMO enzymes outside xenobiotic metabolism is not well understood, but they are induced by various redox stressors and are important for resistance to reductive stress, which affects endoplasmic reticulum protein homeostasis [68, 81, 82]. One proposed function of FMOs may be to counterbalance GSH-mediated redox buffering to promote an oxidative redox environment through O_2_- and NADPH-dependent oxidation of biological thiols [81–83]. Adding to the complexity of FMOs, we observed that both deletion and overexpression of *fmo-2* extend lifespan at 25°C. Interestingly, HIF-1 shows a similar effect on longevity at 25°C, where both deletion and hyperactivation of HIF-1 extend lifespan [84]; these observations could be linked since *fmo-2* is a target of HIF-1 [68]. In the case of *hif-1* deletion at 25°C, lifespan extension requires *daf-16* [84], demonstrating that longevity pathways compensate for each other to regulate organismal stress resistance and aging. Our data are consistent with the model that *tald-1(RNAi)* lifespan extension requires *fmo-2*, but we acknowledge that other factors downstream of either *fmo-2* deletion (i.e. longevity factors induced by reduced *fmo-2* expression) or HLH-30 could also be responsible.

One intriguing twist to this model is that, unlike either dietary restriction [60, 85] or activation of the hypoxic response [86], *tald-1(RNAi)* must occur during development in order to promote longevity. This is similar to the mitochondrial longevity mutants, which have previously been thought to be largely mechanistically distinct from these other longevity pathways. HIF-1 is known to be activated in some long-lived mitochondrial mutants in response to ROS and to mediate part of their lifespan extension [20]; however, HIF-1 is not required for lifespan extension from *tald-1(RNAi)*. Thus, our data suggest that the PPP mediates a complex interaction between several portions of the overall longevity network in worms that have previously been studied as genetically distinct “pathways”. These interactions will be of interest for future studies of longevity and aging in *C*. *elegans*.

Given the highly conserved nature of the PPP and its interactions with cellular metabolism, redox balance, and stress resistance, it is interesting to consider the extent to which the observations reported here will translate to mammals. As previously mentioned, there is good reason to believe that transaldolase deficiency can similarly impact mitochondrial function, metabolism, and oxidative stress resistance in mammals. To the best of our knowledge, there are no reports of PPP or transaldolase inhibition extending lifespan in a mammal; however, the downstream effectors of *tald-1(RNAi)* in worms are likely to play a conserved role in aging, as numerous studies have implicated autophagy in mammalian aging [87] and FMO-2 orthologs are among the most consistently induced enzymes in numerous long-lived mouse models [88, 89].

In summary, we uncovered a novel role of the PPP not only as a central metabolic pathway, but also as a signaling hub that connects the UPR^mt^, p38 and JNK MAPK signaling, and a starvation response mediated by HLH-30 and FMO-2 to promote cellular homeostasis and organismal longevity.

## Materials and methods

### Strains

RB967 (*gcn-2(ok871*)), ZG31 (*hif-1(ia4)*), CF1038 (*daf-16(mu86)*), CB4037 (*glp-1(e2141)*), VC8 (*jnk-1(gk7)*), KB3 (*kgb-1(um3)*), KU25 (*pmk-1(km25)*), VC1668 (*fmo-2(ok2147*)), STE68 (*nhr-49(nr2041)*), VC1024 (*pdr-1(gk448)*), SJ4100 (zcIs13[*hsp-6*_p_::*gfp*]), SJ4143 (zcIs17[*ges-1p*::*gfp*^*mt*^]), BX113 (waEx15 [*fat-7p*::*gfp* + *lin-15*(+)]), MAH235 (sqIs19 [*hlh-30p*::*hlh-30*::*gfp* + *rol-6(su1006)*]), KAE9 (*eft-3p*::*fmo-2* + *h2b*::*gfp* + Cbr-*unc-119*(+)), and VS20 (hjIs67 [*atgl-1p*::*atgl-1*::*gfp* + *mec-7*::*rfp*]) were obtained from the Caenorhabditis Genetics Center (Minneapolis, MN). The *atfs-1(tm4525)* and *hlh-30(tm1978)* strains were obtained from the National BioResource Project (Tokyo, Japan).

The *fmo-2p*::*mCherry* reporter strain, a transcriptional reporter, was created by microinjecting RBW6699 worms with a solution of 50ng/μL of the BSP190 construct containing 2076 bp of genomic sequence preceding the ATG of the *fmo-2* coding sequence followed by the mCherry coding sequence and the unc-54 3’ UTR. A single copy insertion was generated at the chromosome II *ttTi5605* locus using the Mos1 mediated Single Copy transgene Insertion (MosSCI) protocol [90].

### Fluorescence and confocal microscopy

Fluorescence microscopy was performed using Zeiss SteREO Lumar.V12 and Nikon Eclipse E600 microscopes. Worms were immobilized using sodium azide, mounted onto 3% agarose pads, and imaged within a few minutes for reporter experiments. Levamisole was avoided for imaging *hlh-30p*::*hlh-30*::*gfp* animals, since it caused rapid HLH-30 nuclear localization. For reporter assays worms were developed on RNAi bacteria at 20°C and imaged on day 1 of adulthood, except for *fmo-2* reporter experiments, where day 2 adults were imaged. At least three independent experiments with approximately 10 animals per condition per experiment were performed for each reporter with similar results. Statistical analysis for quantification of reporters was performed using student’s t-test with Bonferroni’s correction or ANOVA with Bonferroni’s post-hoc, * *p*<0.05, ** *p*<0.01, *** *p*<0.001.

Confocal microscopy was performed using the Zeiss 510 META Confocal (for imaging mitochondrial morphology) or Leica SP8X (for imaging the HyPer reporter). For imaging intestinal mitochondrial morphology, animals were immobilized using levamisole and mounted on 10% agarose pads to prevent movement during image acquisition. Mitochondria were imaged with a 100X oil objective and Z-stacks of the posterior intestinal cells were taken at 0.34 μm increments. Gain settings for each image were maximized without over-saturation to emphasize mitochondrial content regardless of GFP expression, import, and folding levels. For imaging processing, Z-stacks were deconvoluted using the Iterative Deconvolve 3D plugin in Fiji and 5 image slices were projected using max intensity projection. Mitochondrial content was analyzed by thresholding the 5 image slice projections of different animals for each condition and quantifying the % area of signal within cell boundaries. For quantification, multiple animals for each condition in at least 2 independent experiments were analyzed.

For imaging the HyPer reporter, we removed the animal autofluorescence by performing linear unmixing similar to previous work [36]. Rather than using mean autofluorescence values for age matched animals, however, we took advantage of fluorescence lifetime gating using HyD detectors. By combining the extremely short fluorescence lifetime of the HyPer reporter, and the long autofluorescence lifetime, we were able to use a simple channel subtraction approach to signal unmixing. HyPer transgenic worms were immobilized in 2 mM levamisole on a 3% agarose pad and the anterior of the worm was line-scanned with a 20x objective at 405_ex_500-550_em_ [1.5–5.5 ns], 488_ex_500-550_em_ [1.5–5.5 ns], and 405_ex_450-470_em_ [7–11.5 ns] ("autofluorescence" channel). The fluorescence lifetime of HyPer is extremely short (<4ns), so by using an emission wavelength far from that of HyPer and a much longer lifetime gating [7–11.5ns], the autofluorescence channel contains no signal from HyPer. This simplifies the unmixing problem allowing us to determine R=FAFinHyPerFAFinAF where *R* is ratio of the autofluorescence as measured in each separate HyPer channel to the autofluorescence as measured in the autofluorescence channel. This ratio is determined using age matched wild-type animals fed *EV(RNAi)* and imaging in all three channels. The ratio is then determined by fitting a linear model to values for each pixel in all channels for multiple animals. To ensure that the any variation in imaging parameters or laser function is corrected for, this process is repeated for each of the three experimental groups. The HyPer fluorescence was then determined by removing the autofluorescence contribution to each HyPer channel by the following: *F*_*HyPer*_ = *F*_*HyPer Raw*_ − (*R* * *F*_*AF in AF*_). This allowed the autofluorescence to be removed pixel-by-pixel for each animal. The 488_ex_500-550_em_ channel displayed negligible autofluoresence after fluorescence lifetime gating, so this was only done for the 405_ex_500-550_em_ channel. The final HyPer redox values were determined as $\frac{F_{HyPer\;488Ex}}{F_{HyPer\;405Ex}}$ following autofluorescence removal. For each worm the head was manually outlined, and the focal plane (z-slice) with the greatest combined fluorescence within the head was used for quantification. Intensity normalized ratiometric (INR) images were generated as previously described [36].

### Lifespan analyses

Synchronized eggs or L1 larvae were grown on NGM plates containing 4 mM IPTG, 25 μg/ml carbenicillin and seeded with RNAi bacteria. At the L4/young adult stage, worms were transferred to plates with 50 μM FUDR to prevent hatching of progeny. When necessary, worms were transferred to new plates with fresh bacteria. Lifespans were performed at 25°C for the majority of experiments, unless otherwise noted in the text and figures. Cohorts were examined every 1–3 days using tactile stimulation to verify viability of animals. Animals that displayed vulval rupture were included in analysis, since it is an age-related phenotype [84, 91]. Animals lost due to foraging or bagging were not included in the analysis. All lifespan analyses were replicated using independent cohorts on different dates with replicate statistics provided in S1 Table. *p*-values were calculated using the Wilcoxon rank-sum test.

### Seahorse bioscience respiration assay

To measure *in vivo* oxygen consumption in *C*. *elegans*, we utilized the Seahorse X24 Bioanalyzer (Seahorse Biosciences) as previously described [30, 92]. Worms were grown on concentrated RNAi bacteria (0.15 g/ml) for 3 days at 20°C starting from the L1 stage, washed from plates, and rinsed from bacteria with M9 buffer 4+ times, before being placed in Seahorse XF24 Cell Culture Microplates for analysis. Basal respiration for each condition was analyzed using the average respiration of 5 well replicates over the course of one hour. Respiration for each genotype was measured in at least 4 independent experiments.

### Mitochondrial isolation and clark electrode assays

To measure activity of the ETC, mitochondria were isolated from *C*. *elegans* treated with RNAi bacteria as previously described [93, 94]. To ensure sufficient material for mitochondrial isolations, worm populations were grown for three generations at 20°C. Initially, animals were grown on concentrated RNAi bacteria for two generations and then transferred into 4–6 250 ml liquid cultures. Liquid cultures were propagated for 4–5 days depending on condition and monitored for developmental progression of animals and bacterial density (maintained at ~2 x 10^10^ cells/ml) to avoid starvation. Animals treated with *cco-1(RNAi)* were grown for two generations on *EV(RNAi)* bacteria and then transferred to liquid cultures containing *cco-1(RNAi)*, due to developmental and fecundity issues associated with multiple generations of *cco-1(RNAi)*. Respiration of isolated mitochondria was measured in 4 independent experiments for each condition.

### H_2_O_2_ assay

Measurement of *in vivo* H_2_O_2_ levels was performed using the transgenic HyPer reporter as previously described [36]. Worms were grown on concentrated RNAi bacteria for 4 days starting from L1 (due to growth delay of this strain), washed from plates, and rinsed from bacteria with M9 buffer. At least 3 replicates of 1,000 worms for each condition were pipetted into a black flat bottom 96-well plate. N2 animals grown on *EV(RNAi)* were used as a background control. Fluorescence measurements were made using a BioTek Synergy H1M plate reader.

### Fat staining

Oil Red O staining and analysis was performed as previously described [95]. To quantify fat staining for each condition photos were converted to RGB color, a pseudo flat field correction was applied, images were separated into their respective RGB channels, and fat staining was thresholded in the green channel consistently across all images for a particular experiment. Fat content for each worm was quantified using the integrated density (limited to thresholded signal) of a 40 pixel diameter circle placed below the pharynx (i.e. over the anterior intestinal cells). Two independent experiments were obtained for quantification.

### LC-MS of NAD^+^ and related metabolites

Levels of NAD^+^, NADH, NADP, and NADPH were determined via Ultra Performance Liquid Chromatography coupled with Mass Spectrometry as previously described [96] with some modifications. Briefly, L4 worms were homogenized in 20% HEPES-buffered methanol (pH 7.5) on dry ice. 5 μL of the extract was separated on a BEHAmide column (Waters, Milford MA) using a Acquity UPLC (Waters) and analyzed with a Xevo TQ (Waters, Milford MA) in multiple reaction monitoring mode (MRM). LC solvents were A: H_2_O with 10 mM Ammonium Acetate and 0.1% NH_4_OH, and B: 95:5 Acetonitrile H_2_O with 10 mM Ammonium Acetate and 0.1% NH_4_OH (Alkaline Gradient) for all metabolites. Unique transitions for each metabolite were employed as described previously [96]. The gradient was as in Table 1.

### Quantitative RT–PCR

RNA was isolated from young adult worms using a TRIzol (Life Technologies) chloroform extraction and cDNA was prepared using iScript Reverse Transcription Supermix for qRT-PCR (Bio-Rad). qRT-PCR was used to measure the expression levels of target genes (iTaq Universal SYBR Green Supermix, Bio-Rad) and normalization controls *pmp-3* and *cdc-42* (TaqMan Gene Expression Assays, Life Technologies). The relative standard curve method was used to calculate gene expression. Primers of target genes are listed in S2 Table.

### Western blotting

Protein was isolated from young adult/adult day 1 worms by flash freezing worm pellets in liquid nitrogen followed by extraction in lysis buffer [20 mM HEPES, pH 7.4, 150 mM NaCl, 1 mM EDTA, 1 mM EGTA, 1% (v/v) Triton X-100, and 1x Pierce Protease Inhibitor Mini Tablets, EDTA Free (88666, ThermoFisher Scientific)]. Proteins of interest were detected by immunoblot using anti-GFP (sc-9996; Santa Cruz Biotechnology), anti-p-JNK (Cell Signaling Technology), anti-p-p38 (Cell Signaling Technology), anti-p-KGB-1 (a gift from Drs. Naoki Hisamoto and Kunihiro Matsumoto), and anti-alpha-tubulin (Clone: DM1A, MS-581-P0, Neomarkers) antibodies at a 1:1000 dilution in 5% BSA TBS-T.

## Supporting information

S1 Fig Pentose phosphate pathway inhibition induces the UPR^mt^ and extends lifespan independent of *atfs-1*. **(A)** Mean relative fluorescence of *hsp-6p*::*gfp* animals grown on PPP RNAi. Fluorescence is calculated relative to *EV(RNAi)* controls (N = 4 independent experiments, pooled individual worm values, error bars indicate s.e.m., student’s t-test with Bonferroni’s correction). **(B)** N2 fed *EV(RNAi)* (mean 21.2±0.3 days, n = 273), N2 fed *tald-1(RNAi)* (mean 24±0.3 days, n = 289), *atfs-1(gk3094)* fed *EV(RNAi)* (mean 19.4±0.4 days, n = 245), *atfs-1(gk3094)* fed *tald-1(RNAi)* (mean 23.7±0.3 days, n = 304). Lifespans were performed at 20°C, with pooled data from two independent experiments shown. **(C)** N2 fed *EV(RNAi)* (mean 20.9±0.2 days, n = 645), N2 fed *cco-1(RNAi)* (mean 30.6±0.3 days, n = 676), *atfs-1(gk3094)* fed *EV(RNAi)* (mean 19±0.3 days, n = 512), *atfs-1(gk3094)* fed *cco-1(RNAi)* (mean 26.2±0.5 days, n = 470). Lifespans were performed at 20°C, with pooled data from five independent experiments shown.
(TIF)

S2 Fig RNAi knockdown of *tald-1(RNAi)* extends lifespan independent of *daf-16*, *aak-2*, *glp-1*, and *hif-1*. **(A)** N2 fed *EV(RNAi)* (mean 15.4±0.2 days, n = 261), N2 fed *tald-1(RNAi)* (mean 18.2±0.2 days, n = 267), *daf-16(mu86)* fed *EV(RNAi)* (mean 12.9±0.1 days, n = 255), *daf-16(mu86)* fed *tald-1(RNAi)* (mean 14.6±0.1 days, n = 263). Lifespans were performed at 25°C, with pooled data from three independent experiments shown. **(B)** N2 fed *EV(RNAi)* (mean 16.3±0.2 days, n = 283), N2 fed *tald-1(RNAi)* (mean 19.6±0.2 days, n = 293), *aak-2(ok524)* fed *EV(RNAi)* (mean 14.3±0.1 days, n = 358), *aak-2(ok524)* fed *tald-1(RNAi)* (mean 16.7±0.1 days, n = 293). Lifespans were performed at 25°C, with pooled data from three independent experiments shown. **(C)** N2 fed *EV(RNAi)* (mean 16±0.1 days, n = 331), N2 fed *tald-1(RNAi)* (mean 18.2±0.2 days, n = 433), *glp-1(e2141)* fed *EV(RNAi)* (mean 18.1±0.1 days, n = 385), *glp-1(e2141)* fed *tald-1(RNAi)* (mean 22.3±0.1 days, n = 359). Lifespans were performed at 25°C, with pooled data from three independent experiments shown. **(D)** N2 fed *EV(RNAi)* (mean 16.5±0.1 days, n = 303), N2 fed *tald-1(RNAi)* (mean 18.9±0.2 days, n = 317), *hif-1(ia4)* fed *EV(RNAi)* (mean 17.1±0.2 days, n = 335), *hif-1(ia4)* fed *tald-1(RNAi)* (mean 20.4±0.2 days, n = 328). Lifespans were performed at 25°C, with pooled data from three independent experiments shown. **(E)** N2 fed *EV(RNAi)* (mean 15.5±0.1 days, n = 328), N2 fed *cco-1(RNAi)* (mean 22±0.2 days, n = 359), *hif-1(ia4)* fed *EV(RNAi)* (mean 16.2±0.1 days, n = 330), *hif-1(ia4)* fed *cco-1(RNAi)* (mean 18.6±0.2 days, n = 352). Lifespans were performed at 25°C, with pooled data from three independent experiments shown. Lifespans in this figure are indicated as mean±s.e.m. and statistical analysis is provided in S1 Table.
(TIF)

S3 Fig Transaldolase deficiency does not alter whole-animal mtDNA content. **(A)** mtDNA content (*nd-1/act-3* DNA) in L4 *tald-1(RNAi)* or *cco-1(RNAi)* animals does not change (n = 15 animals, error bars indicate s.e.m., student’s t-test with Bonferroni’s correction). **(B)** mtDNA content (*nd-1/act-3* DNA) in adult day 1 *tald-1(RNAi)* or *cco-1(RNAi)* animals does not change (n = 16 animals, error bars indicate s.e.m., student’s t-test with Bonferroni’s correction).
(TIF)

S4 Fig Transaldolase deficiency causes a mitochondrial morphology shift independent of *pdr-1*, *pink-1*, and *fzo-1*. **(A)** RNAi knockdown of mitochondrial fusion and fission factors alters intestinal mitochondrial morphology. *ges-1p*::*gfp*^*mt*^ reporter animals were imaged and max intensity projections of five z-slices are presented. RNAi knockdown of *tald-1* alters mitochondrial morphology independent of **(B)** *pdr-1*, **(C)** *fzo-1*, and *pink-1*. For (B), *pdr-1(gk448)* mutants were used. *ges-1p*::*gfp*^*mt*^ reporter animals were imaged and max intensity projections of five z-slices are presented. Scale bar, 10 μm.
(TIF)

S5 Fig Length and density of *tald-1(RNAi)* and *cco-1(RNAi)* animals. RNAi knockdown of *cco-1*, but not *tald-1* reduces the **(A)** extinction coefficient and **(B)** time of flight of *C*. *elegans*. N2 animals were grown on RNAi bacteria for 3 days from hatching, washed off plates, and analyzed using the COPAS BIOSORT. In this figure, statistics are displayed as: * *p*<0.05, ** *p*<0.01, *** *p*<0.001.
(TIF)

S6 Fig Transaldolase deficiency increases oxidation of the HyPer reporter. **(A)** Representative INR images of HyPer animals grown on *tald-1(RNAi)* or *cco-1(RNAi)*. Two images for each condition are shown to emphasize variability in HyPer oxidation across individual worms and consistent effects of *tald-1(RNAi)* on oxidation of the reporter. **(B)** Confocal image quantification of HyPer reporter (N = 3 independent experiments, pooled individual worm values, error bars indicate s.e.m., student’s t-test with Bonferroni’s correction). In this figure, statistics are displayed as: * *p*<0.05.
(TIF)

S7 Fig JNK MAPKs do not alter mitochondrial dysfunction from *tald-1(RNAi)* or *cco-1(RNAi)* and are not required for *daf-2(RNAi)* longevity. **(A)** JNK-1 is not required for *daf-2(RNAi)* lifespan extension. N2 fed *EV(RNAi)* (mean 17.4±0.1 days, n = 313), N2 fed *daf-2 (RNAi)* (mean 26.1±0.2 days, n = 272), *jnk-1(gk7)* fed *EV(RNAi)* (mean 17.6±0.1 days, n = 363), *jnk-1(gk7)* fed *daf-2(RNAi)* (mean 26±0.2 days, n = 332). Lifespans were performed at 25°C, with pooled data from three independent experiments shown. **(B)** KGB-1 is not required for *daf-2(RNAi)* lifespan extension. N2 fed *EV(RNAi)* (mean 15±0.1 days, n = 630), N2 fed *daf-2 (RNAi)* (mean 23.4±0.2 days, n = 633), *kgb-1(um3)* fed *EV(RNAi)* (mean 13.1±0.1 days, n = 580), *kgb-1(um3)* fed *daf-2(RNAi)* (mean 25.5±0.2 days, n = 563). Lifespans were performed at 25°C, with pooled data from four independent experiments shown. **(C)** *hsp-6p*::*gfp* reporter induction in *tald-1(RNAi)* or *cco-1(RNAi)* animals is not prevented from *jnk-1(gk7) and kgb-1(um3)* mutations. Scale bar, 200 μm. **(D)** Mean relative fluorescence of *hsp-6p*::*gfp* reporter animals. Fluorescence is calculated relative to N2 *EV(RNAi)* controls (N = 2 independent experiments, pooled individual worm values, error bars indicate s.e.m., student’s t-test with Bonferroni’s correction). **(E)** Oxygen consumption rate decreases independent of JNK-1 and KGB-1 from *tald-1(RNAi)* or *cco-1(RNAi)*. OCR was measured using the Seahorse XF Analyzer and normalized to animal number (N = 6 independent experiments for N2 animals, N = 4 independent experiments for *jnk-1(gk7)* and *kgb-1(um3)* animals, error bars indicate s.e.m., student’s t-test with Bonferroni’s correction). Lifespans in this figure are indicated as mean±s.e.m. and statistical analysis is provided in S1 Table. In this figure, statistics are displayed as: * *p*<0.05, ** *p*<0.01, *** *p*<0.001.
(TIF)

S8 Fig NSY-1/PMK-1 is not required for UPR^mt^ induction from *tald-1(RNAi)* or *cco-1(RNAi)*, but is required for *daf-2(RNAi)* longevity. **(A)** *hsp-6p*::*gfp* reporter induction in *tald-1(RNAi)* or *cco-1(RNAi)* animals is not prevented by the *pmk-1(km25)* mutation. **(B)** Mean relative fluorescence of *hsp-6p*::*gfp* reporter animals in the context of the *pmk-1(km25)* mutation. Fluorescence is calculated relative to N2 *EV(RNAi)* controls (N = 2 independent experiments, pooled individual worm values, error bars indicate s.e.m., student’s t-test with Bonferroni’s correction). **(C)** PMK-1 is partially required for *daf-2(RNAi)* lifespan extension. N2 fed *EV(RNAi)* (mean 16.7±0.1 days, n = 423), N2 fed *daf-2 (RNAi)* (mean 26.3±0.3 days, n = 325), *pmk-1(km25)* fed *EV(RNAi)* (mean 14.6±0.1 days, n = 385), *pmk-1(km25)* fed *daf-2(RNAi)* (mean 19.8±0.3 days, n = 295). Lifespans were performed at 25°C, with pooled data from three independent experiments shown. **(D)** NSY-1 is partially required for *daf-2(RNAi)* lifespan extension. N2 fed *EV(RNAi)* (mean 14.6±0.1 days, n = 542), N2 fed *daf-2 (RNAi)* (mean 23.1±0.2 days, n = 544), *nsy-1(ag3)* fed *EV(RNAi)* (mean 14.9±0.1 days, n = 473), *nsy-1(ag3)* fed *daf-2(RNAi)* (mean 20.7±0.3 days, n = 480). Lifespans were performed at 25°C, with pooled data from four independent experiments shown. **(E)** MAPKs in *C*. *elegans* are activated by H_2_O_2_ treatment. Western blot analysis was performed on protein lysates isolated from animals exposed to 10 mM H_2_O_2_ for either 0, 5, 15, 30, or 60 minutes in M9 media. Lifespans in this figure are indicated as mean±s.e.m. and statistical analysis is provided in S1 Table. In this figure, statistics are displayed as: * *p*<0.05, ** *p*<0.01, *** *p*<0.001.
(TIF)

S9 Fig RNAi knockdown of *cco-1*, but not *tald-1* reduces pharyngeal pumping rate in young animals. **(A)** Pumping rate per minute was measured for individual animals, with each dot representing an individual (N = 3 independent experiments, error bars indicate s.e.m., student’s t-test with Bonferroni’s correction). In this figure, statistics are displayed as: * *p*<0.05, ** *p*<0.01, *** *p*<0.001.
(TIF)

S10 Fig JNK-1 and KGB-1 are not required for *fmo-2* reporter induction and FMO-2 is not required for *hsp-6* reporter induction. **(A)** RNAi knockdown of *cco-1* increases *fmo-2p*::*mCherry* reporter induction in cells proximal to the anterior bulb. *fmo-2p*::*mCherry* reporter animals were grown on RNAi bacteria from hatching and imaged 4 days later using fluorescent microscopy. **(B)** Mean relative fluorescence of *fmo-2p*::*mCherry* reporter animals in the context of *jnk-1(gk7)* and *kgb-1(um3)* mutations. Fluorescence is calculated relative to N2 *EV(RNAi)* controls (N = 3 independent experiments, pooled individual worm values, error bars indicate s.e.m., ANOVA with Bonferroni’s post-hoc). **(C)** Mean relative fluorescence of *hsp-6p*::*gfp* reporter animals in the context of the *fmo-2(ok2147)* mutation. Fluorescence is calculated relative to N2 *EV(RNAi)* controls (N = 3 independent experiments, pooled individual worm values, error bars indicate s.e.m., student’s t-test with Bonferroni’s correction). In this figure, statistics are displayed as: * *p*<0.05, ** *p*<0.01, *** *p*<0.001.
(TIF)

S1 Table Lifespan data and statistical analyses. *p*-values are shown for condition comparison* or genotype to N2 comparison** and calculated using Wilcoxon Rank-Sum test.
(XLSX)

S2 Table qRT-PCR primer list. Forward and reverse primer sequences are listed for each gene set tested.
(XLSX)
