# Supplementary figures and images for: Transaldolase inhibition impairs mitochondrial respiration and induces a starvation-like longevity response in Caenorhabditis elegans
Source: PLoS Genet. 2017 Mar 29;13(3):e1006695. doi: 10.1371/journal.pgen.1006695 (PMC5389855; doi:10.1371/journal.pgen.1006695)

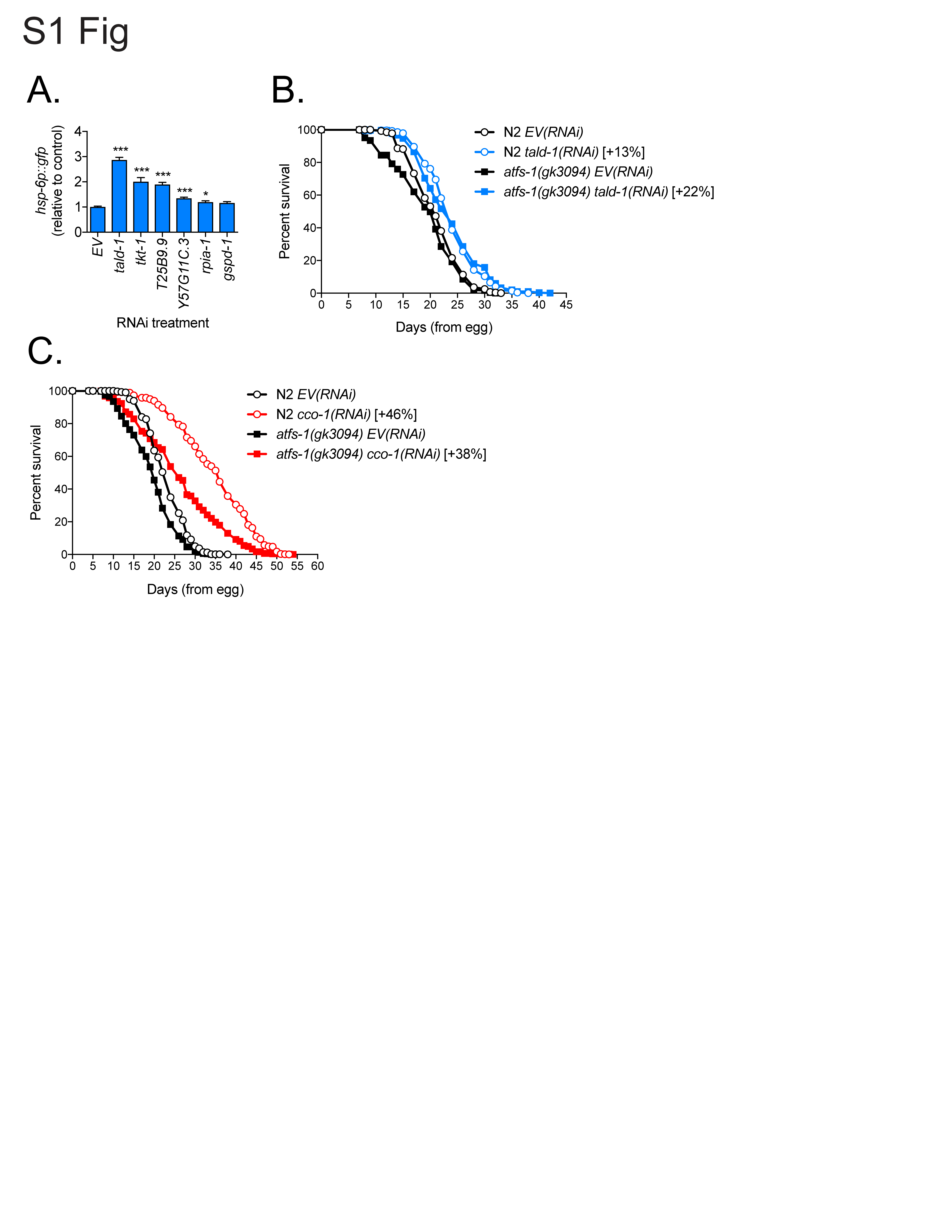

Supplement: S1 Fig — (A) Mean relative fluorescence of hsp-6p::gfp animals grown on PPP RNAi. Fluorescence is calculated relative to EV(RNAi) controls (N = 4 independent experiments, pooled individual worm values, error bars indicate s.e.m., student’s t-test with Bonferroni’s correction). (B) N2 fed EV(RNAi) (mean 21.2±0.3 days, n = 273), N2 fed tald-1(RNAi) (mean 24±0.3 days, n = 289), atfs-1(gk3094) fed EV(RNAi) (mean 19.4±0.4 days, n = 245), atfs-1(gk3094) fed tald-1(RNAi) (mean 23.7±0.3 days, n = 304). Lifespans were performed at 20°C, with pooled data from two independent experiments shown. (C) N2 fed EV(RNAi) (mean 20.9±0.2 days, n = 645), N2 fed cco-1(RNAi) (mean 30.6±0.3 days, n = 676), atfs-1(gk3094) fed EV(RNAi) (mean 19±0.3 days, n = 512), atfs-1(gk3094) fed cco-1(RNAi) (mean 26.2±0.5 days, n = 470). Lifespans were performed at 20°C, with pooled data from five independent experiments shown. (TIF) [file pgen.1006695.s001.tif]

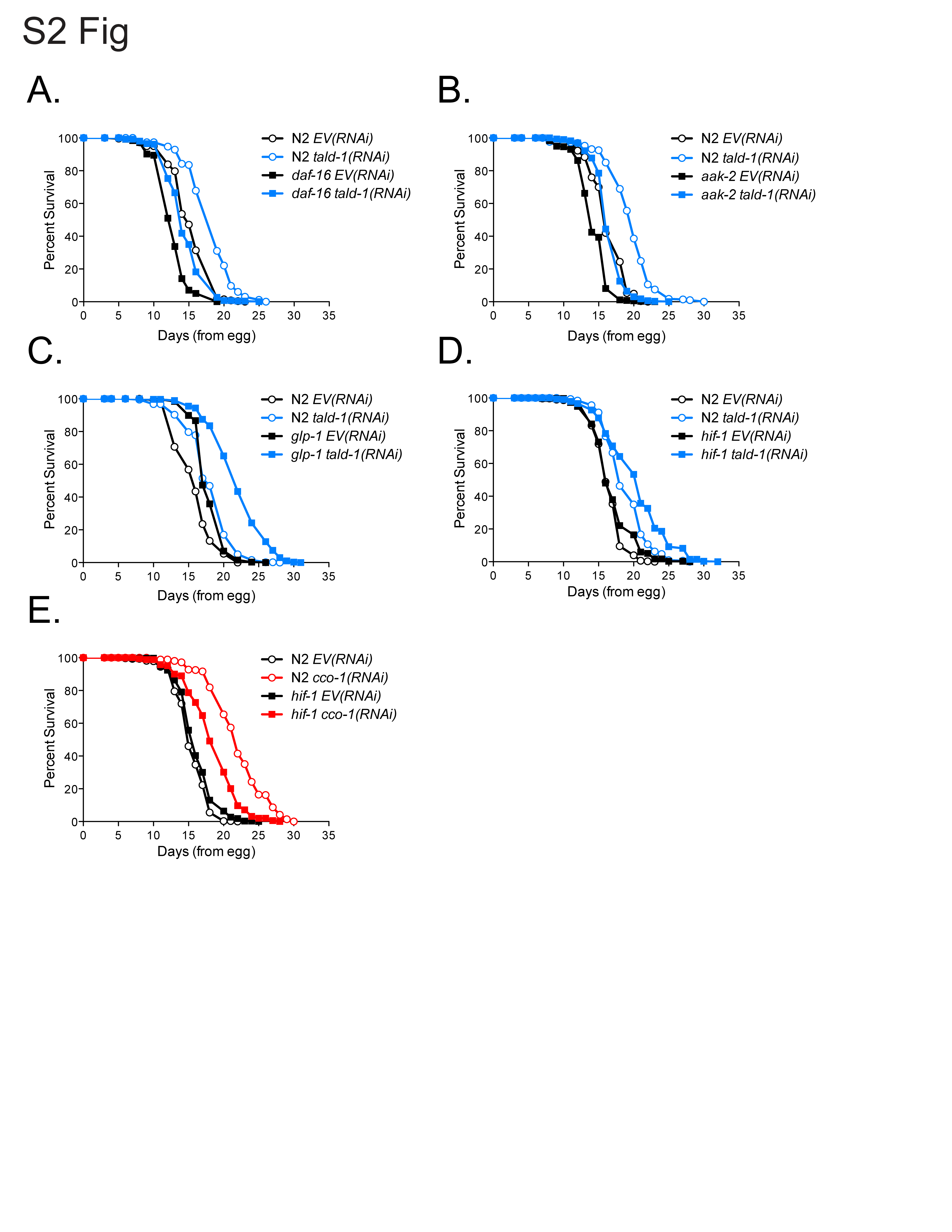

Supplement: S2 Fig — (A) N2 fed EV(RNAi) (mean 15.4±0.2 days, n = 261), N2 fed tald-1(RNAi) (mean 18.2±0.2 days, n = 267), daf-16(mu86) fed EV(RNAi) (mean 12.9±0.1 days, n = 255), daf-16(mu86) fed tald-1(RNAi) (mean 14.6±0.1 days, n = 263). Lifespans were performed at 25°C, with pooled data from three independent experiments shown. (B) N2 fed EV(RNAi) (mean 16.3±0.2 days, n = 283), N2 fed tald-1(RNAi) (mean 19.6±0.2 days, n = 293), aak-2(ok524) fed EV(RNAi) (mean 14.3±0.1 days, n = 358), aak-2(ok524) fed tald-1(RNAi) (mean 16.7±0.1 days, n = 293). Lifespans were performed at 25°C, with pooled data from three independent experiments shown. (C) N2 fed EV(RNAi) (mean 16±0.1 days, n = 331), N2 fed tald-1(RNAi) (mean 18.2±0.2 days, n = 433), glp-1(e2141) fed EV(RNAi) (mean 18.1±0.1 days, n = 385), glp-1(e2141) fed tald-1(RNAi) (mean 22.3±0.1 days, n = 359). Lifespans were performed at 25°C, with pooled data from three independent experiments shown. (D) N2 fed EV(RNAi) (mean 16.5±0.1 days, n = 303), N2 fed tald-1(RNAi) (mean 18.9±0.2 days, n = 317), hif-1(ia4) fed EV(RNAi) (mean 17.1±0.2 days, n = 335), hif-1(ia4) fed tald-1(RNAi) (mean 20.4±0.2 days, n = 328). Lifespans were performed at 25°C, with pooled data from three independent experiments shown. (E) N2 fed EV(RNAi) (mean 15.5±0.1 days, n = 328), N2 fed cco-1(RNAi) (mean 22±0.2 days, n = 359), hif-1(ia4) fed EV(RNAi) (mean 16.2±0.1 days, n = 330), hif-1(ia4) fed cco-1(RNAi) (mean 18.6±0.2 days, n = 352). Lifespans were performed at 25°C, with pooled data from three independent experiments shown. Lifespans in this figure are indicated as mean±s.e.m. and statistical analysis is provided in S1 Table. (TIF) [file pgen.1006695.s002.tif]

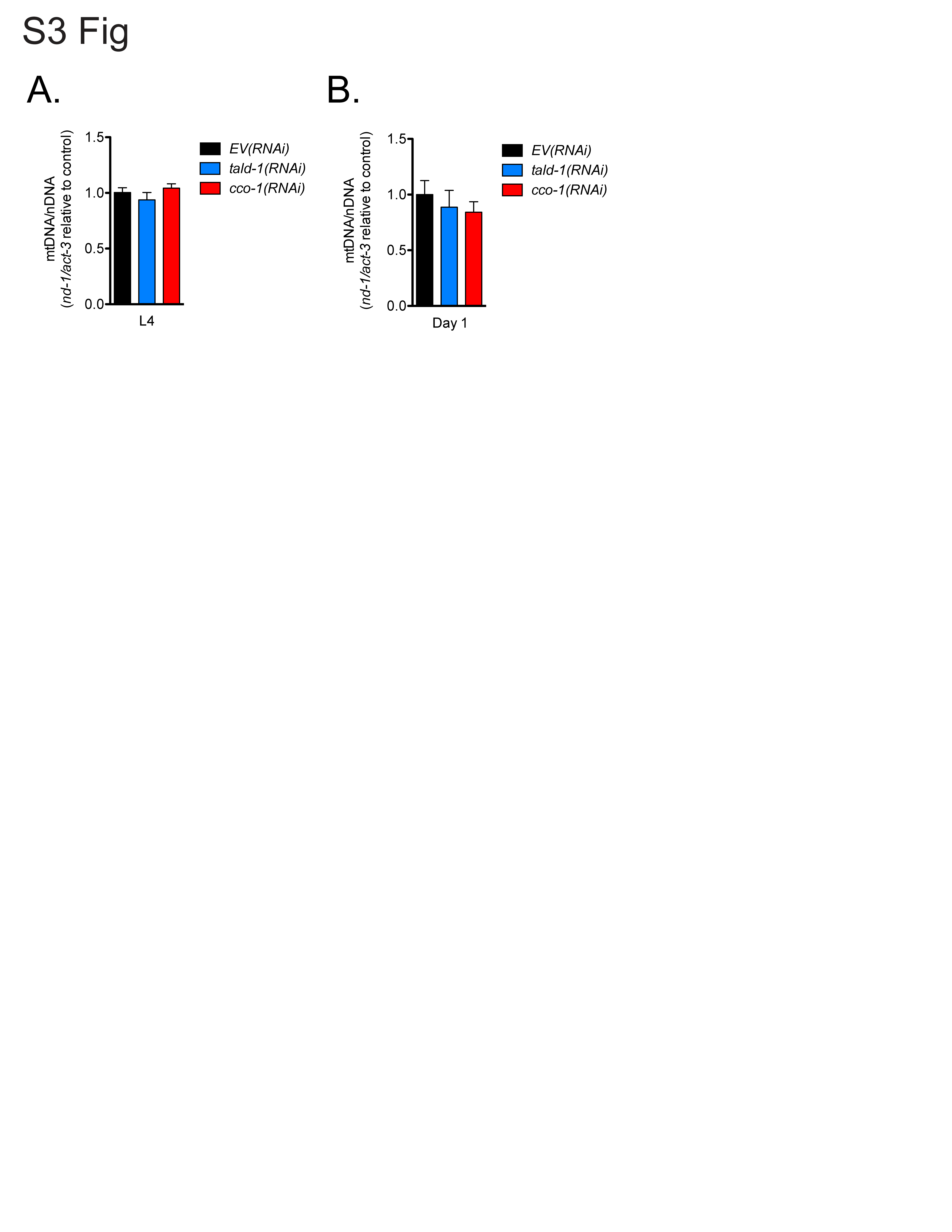

Supplement: S3 Fig — (A) mtDNA content (nd-1/act-3 DNA) in L4 tald-1(RNAi) or cco-1(RNAi) animals does not change (n = 15 animals, error bars indicate s.e.m., student’s t-test with Bonferroni’s correction). (B) mtDNA content (nd-1/act-3 DNA) in adult day 1 tald-1(RNAi) or cco-1(RNAi) animals does not change (n = 16 animals, error bars indicate s.e.m., student’s t-test with Bonferroni’s correction). (TIF) [file pgen.1006695.s003.tif]

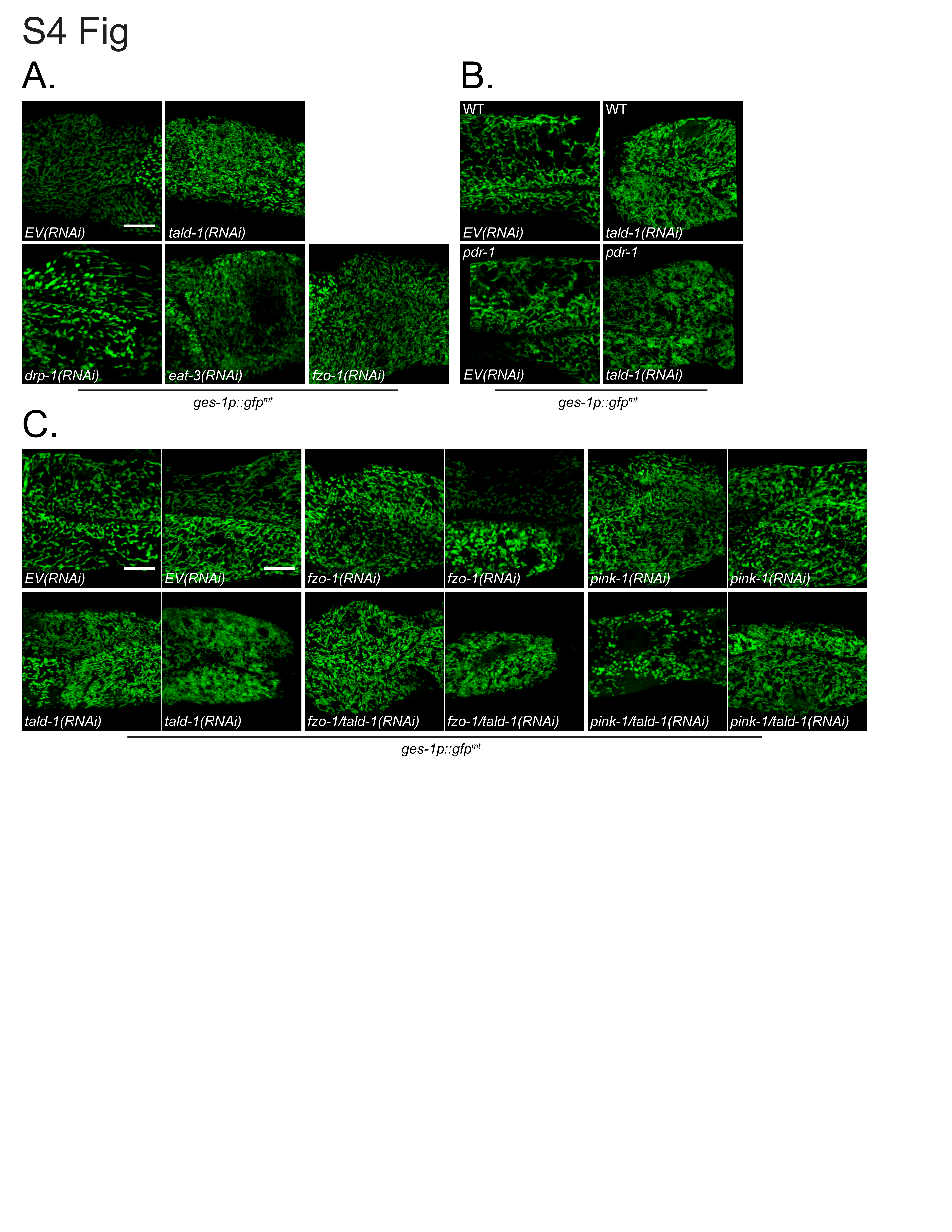

Supplement: S4 Fig — (A) RNAi knockdown of mitochondrial fusion and fission factors alters intestinal mitochondrial morphology. ges-1p::gfpmt reporter animals were imaged and max intensity projections of five z-slices are presented. RNAi knockdown of tald-1 alters mitochondrial morphology independent of (B) pdr-1, (C) fzo-1, and pink-1. For (B), pdr-1(gk448) mutants were used. ges-1p::gfpmt reporter animals were imaged and max intensity projections of five z-slices are presented. Scale bar, 10 μm. (TIF) [file pgen.1006695.s004.tif]

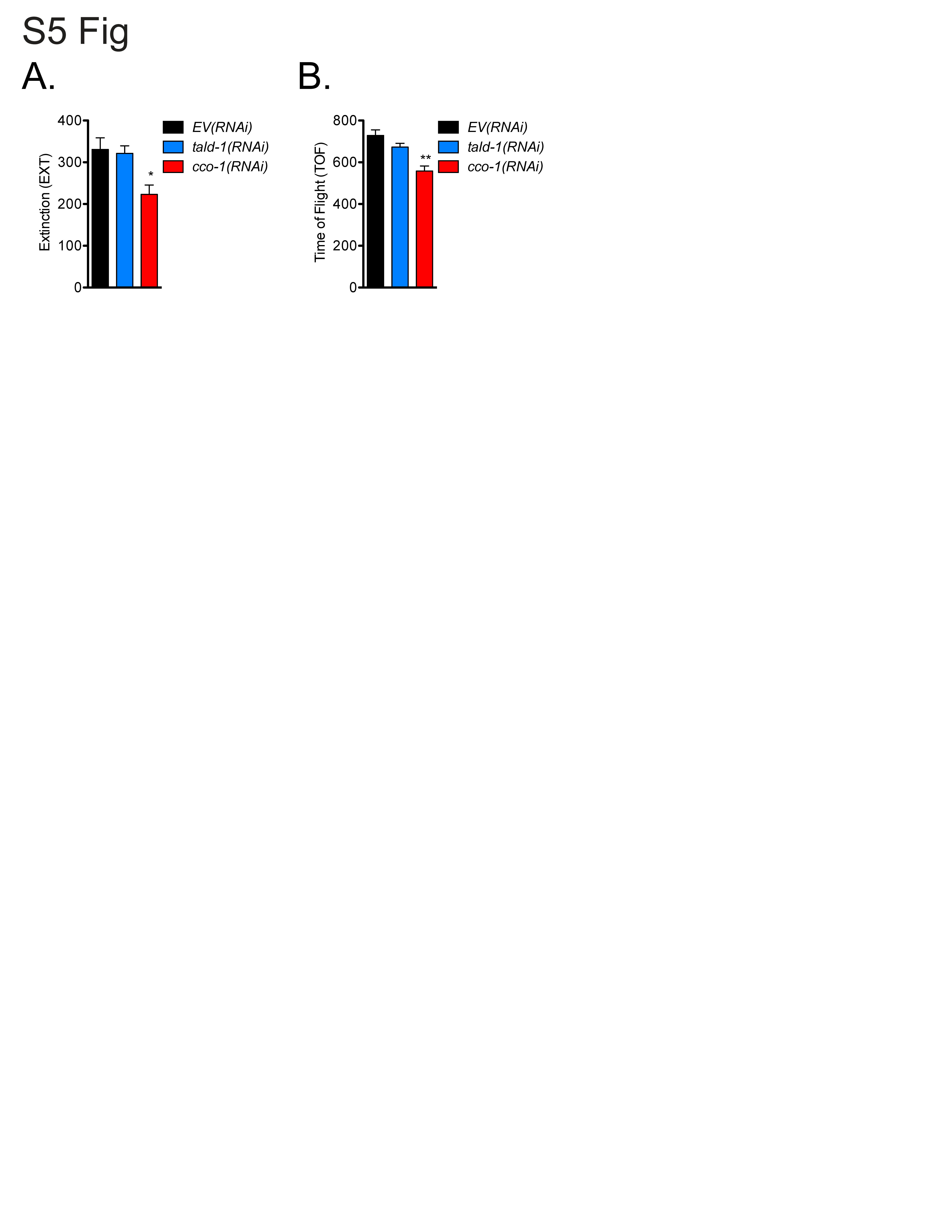

Supplement: S5 Fig — RNAi knockdown of cco-1, but not tald-1 reduces the (A) extinction coefficient and (B) time of flight of C. elegans. N2 animals were grown on RNAi bacteria for 3 days from hatching, washed off plates, and analyzed using the COPAS BIOSORT. In this figure, statistics are displayed as: * p<0.05, ** p<0.01, *** p<0.001. (TIF) [file pgen.1006695.s005.tif]

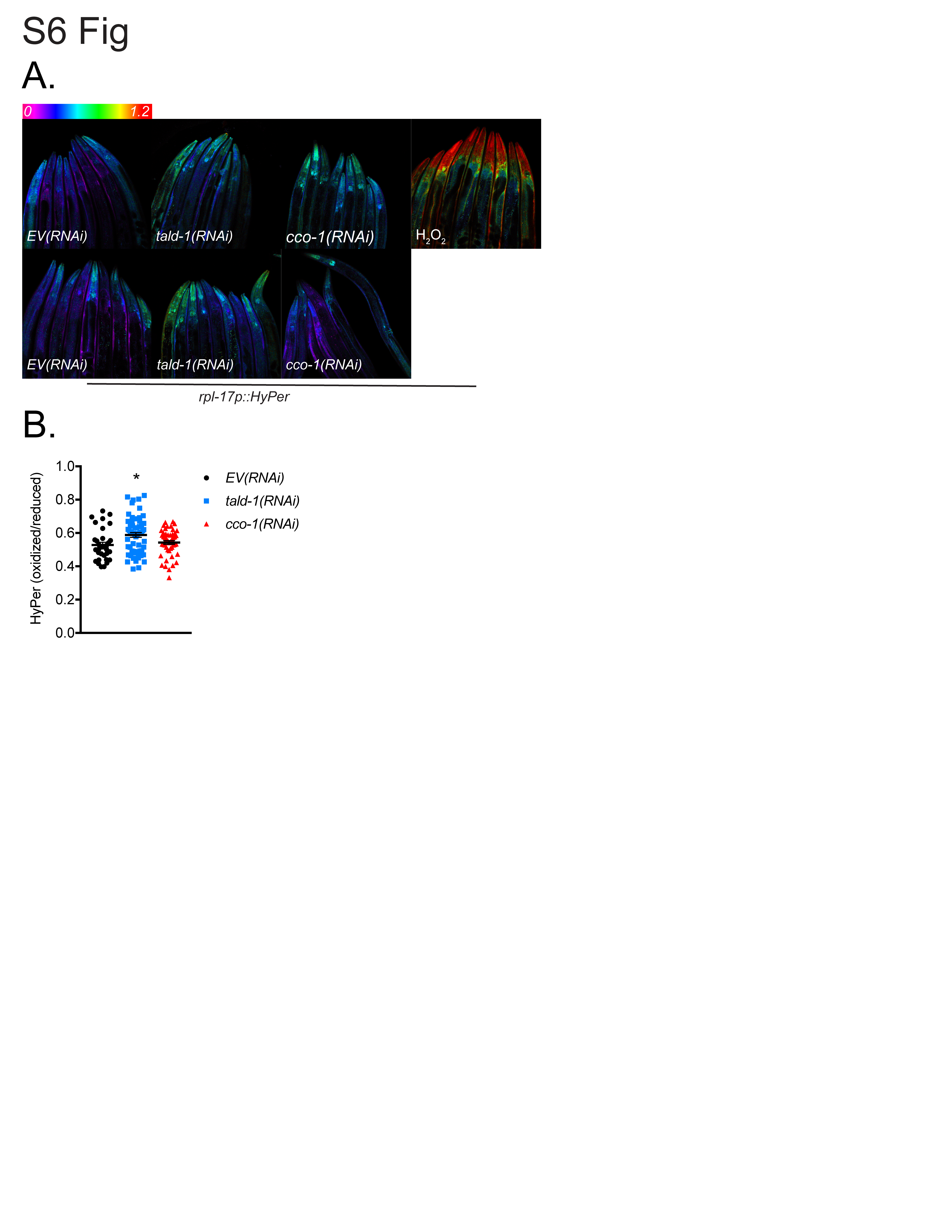

Supplement: S6 Fig — (A) Representative INR images of HyPer animals grown on tald-1(RNAi) or cco-1(RNAi). Two images for each condition are shown to emphasize variability in HyPer oxidation across individual worms and consistent effects of tald-1(RNAi) on oxidation of the reporter. (B) Confocal image quantification of HyPer reporter (N = 3 independent experiments, pooled individual worm values, error bars indicate s.e.m., student’s t-test with Bonferroni’s correction). In this figure, statistics are displayed as: * p<0.05. (TIF) [file pgen.1006695.s006.tif]

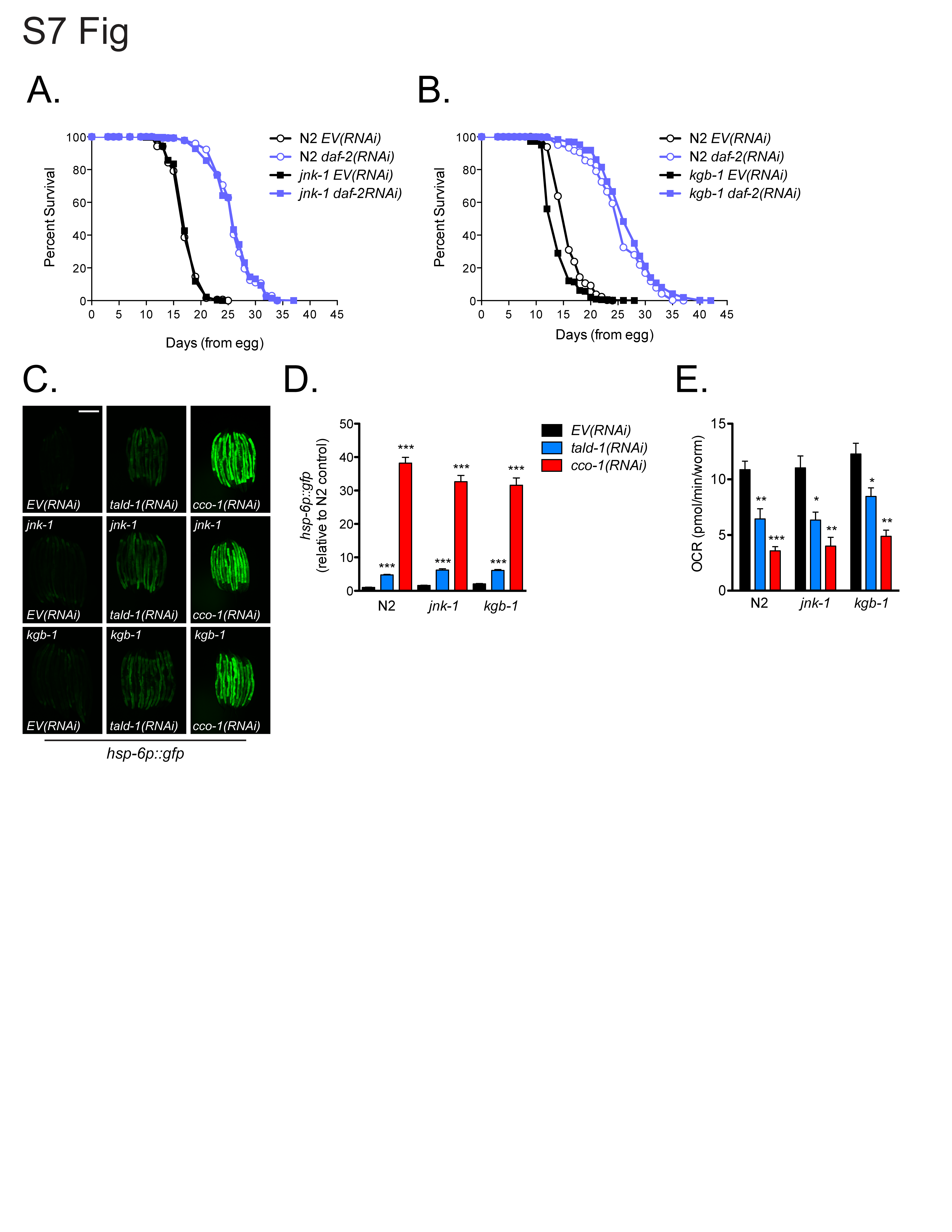

Supplement: S7 Fig — (A) JNK-1 is not required for daf-2(RNAi) lifespan extension. N2 fed EV(RNAi) (mean 17.4±0.1 days, n = 313), N2 fed daf-2 (RNAi) (mean 26.1±0.2 days, n = 272), jnk-1(gk7) fed EV(RNAi) (mean 17.6±0.1 days, n = 363), jnk-1(gk7) fed daf-2(RNAi) (mean 26±0.2 days, n = 332). Lifespans were performed at 25°C, with pooled data from three independent experiments shown. (B) KGB-1 is not required for daf-2(RNAi) lifespan extension. N2 fed EV(RNAi) (mean 15±0.1 days, n = 630), N2 fed daf-2 (RNAi) (mean 23.4±0.2 days, n = 633), kgb-1(um3) fed EV(RNAi) (mean 13.1±0.1 days, n = 580), kgb-1(um3) fed daf-2(RNAi) (mean 25.5±0.2 days, n = 563). Lifespans were performed at 25°C, with pooled data from four independent experiments shown. (C) hsp-6p::gfp reporter induction in tald-1(RNAi) or cco-1(RNAi) animals is not prevented from jnk-1(gk7) and kgb-1(um3) mutations. Scale bar, 200 μm. (D) Mean relative fluorescence of hsp-6p::gfp reporter animals. Fluorescence is calculated relative to N2 EV(RNAi) controls (N = 2 independent experiments, pooled individual worm values, error bars indicate s.e.m., student’s t-test with Bonferroni’s correction). (E) Oxygen consumption rate decreases independent of JNK-1 and KGB-1 from tald-1(RNAi) or cco-1(RNAi). OCR was measured using the Seahorse XF Analyzer and normalized to animal number (N = 6 independent experiments for N2 animals, N = 4 independent experiments for jnk-1(gk7) and kgb-1(um3) animals, error bars indicate s.e.m., student’s t-test with Bonferroni’s correction). Lifespans in this figure are indicated as mean±s.e.m. and statistical analysis is provided in S1 Table. In this figure, statistics are displayed as: * p<0.05, ** p<0.01, *** p<0.001. (TIF) [file pgen.1006695.s007.tif]

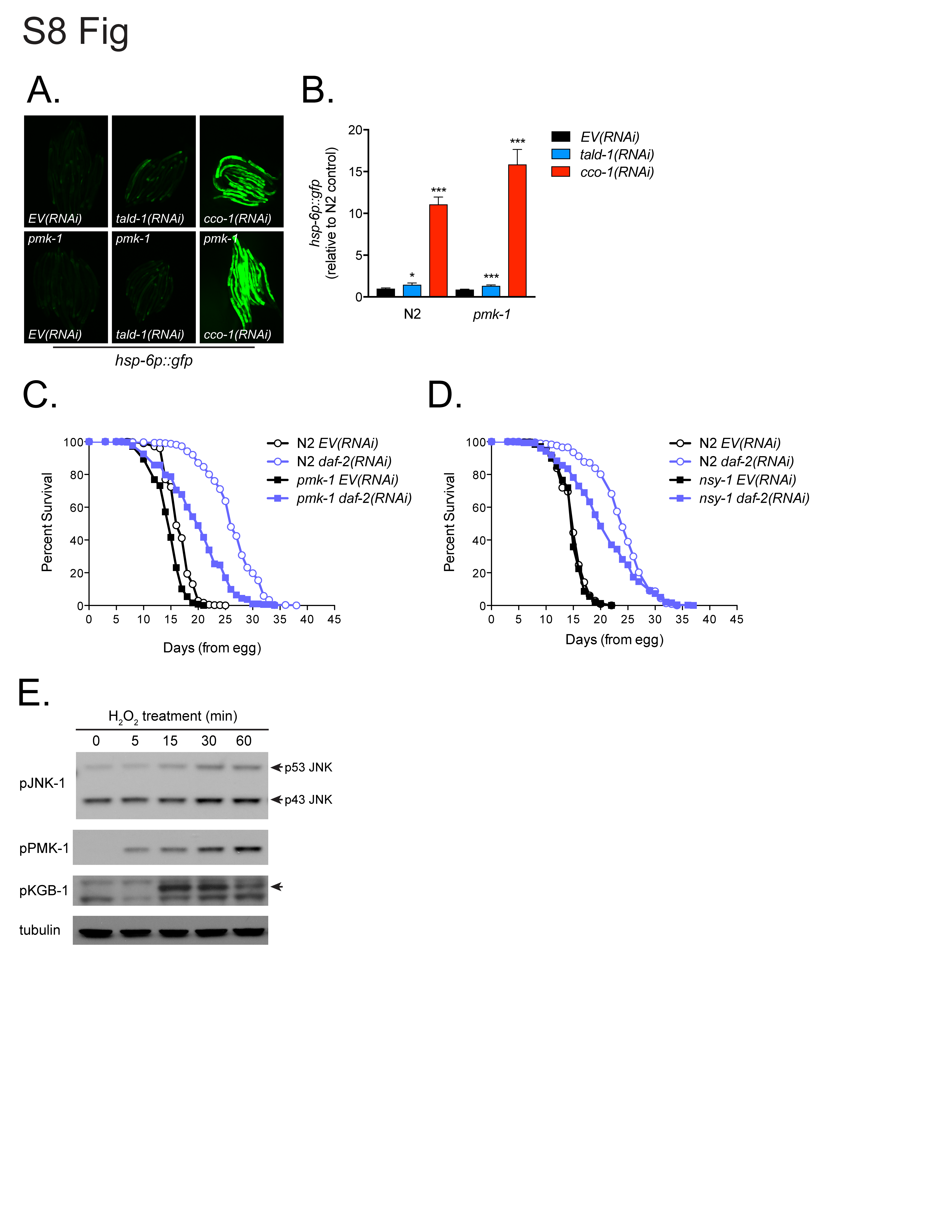

Supplement: S8 Fig — (A) hsp-6p::gfp reporter induction in tald-1(RNAi) or cco-1(RNAi) animals is not prevented by the pmk-1(km25) mutation. (B) Mean relative fluorescence of hsp-6p::gfp reporter animals in the context of the pmk-1(km25) mutation. Fluorescence is calculated relative to N2 EV(RNAi) controls (N = 2 independent experiments, pooled individual worm values, error bars indicate s.e.m., student’s t-test with Bonferroni’s correction). (C) PMK-1 is partially required for daf-2(RNAi) lifespan extension. N2 fed EV(RNAi) (mean 16.7±0.1 days, n = 423), N2 fed daf-2 (RNAi) (mean 26.3±0.3 days, n = 325), pmk-1(km25) fed EV(RNAi) (mean 14.6±0.1 days, n = 385), pmk-1(km25) fed daf-2(RNAi) (mean 19.8±0.3 days, n = 295). Lifespans were performed at 25°C, with pooled data from three independent experiments shown. (D) NSY-1 is partially required for daf-2(RNAi) lifespan extension. N2 fed EV(RNAi) (mean 14.6±0.1 days, n = 542), N2 fed daf-2 (RNAi) (mean 23.1±0.2 days, n = 544), nsy-1(ag3) fed EV(RNAi) (mean 14.9±0.1 days, n = 473), nsy-1(ag3) fed daf-2(RNAi) (mean 20.7±0.3 days, n = 480). Lifespans were performed at 25°C, with pooled data from four independent experiments shown. (E) MAPKs in C. elegans are activated by H2O2 treatment. Western blot analysis was performed on protein lysates isolated from animals exposed to 10 mM H2O2 for either 0, 5, 15, 30, or 60 minutes in M9 media. Lifespans in this figure are indicated as mean±s.e.m. and statistical analysis is provided in S1 Table. In this figure, statistics are displayed as: * p<0.05, ** p<0.01, *** p<0.001. (TIF) [file pgen.1006695.s008.tif]

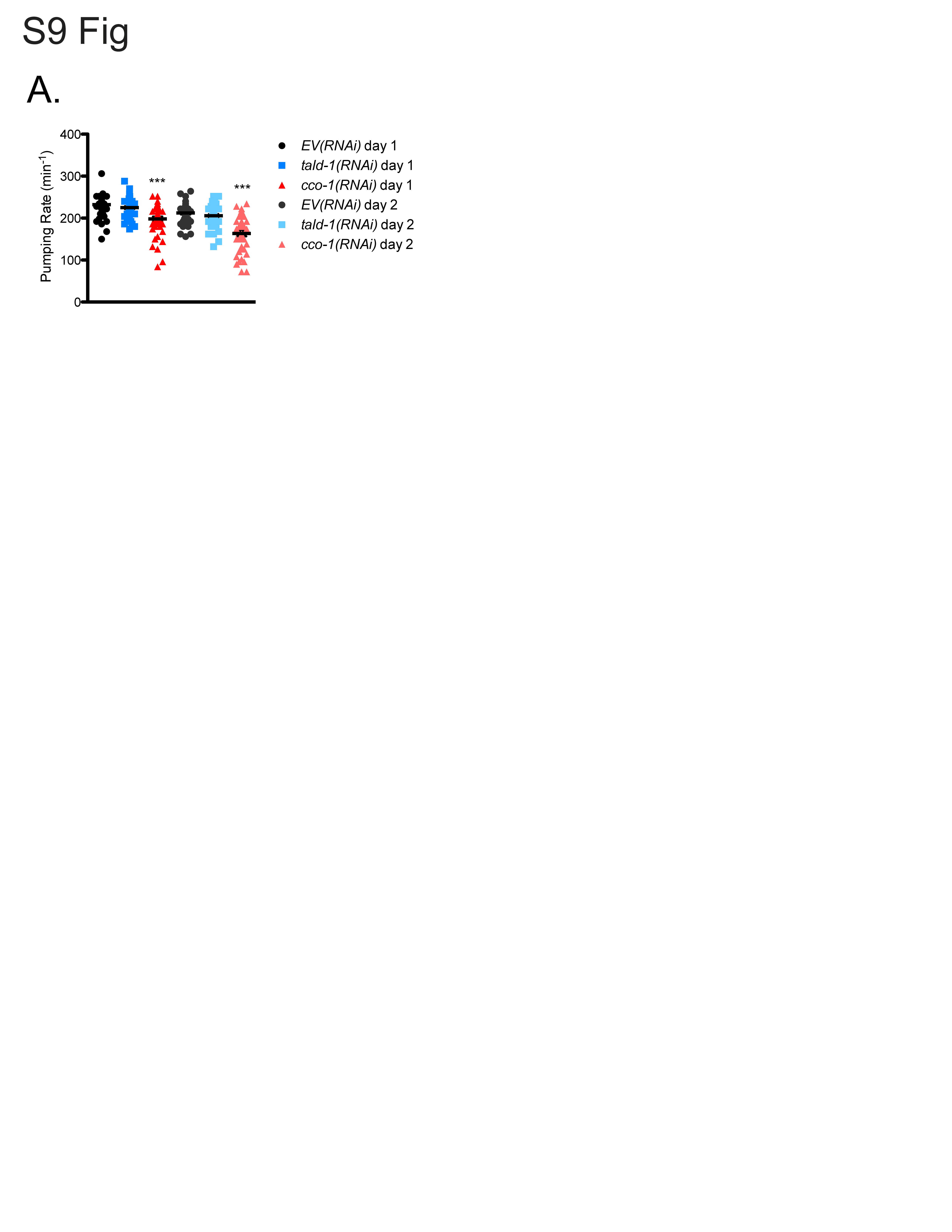

Supplement: S9 Fig — (A) Pumping rate per minute was measured for individual animals, with each dot representing an individual (N = 3 independent experiments, error bars indicate s.e.m., student’s t-test with Bonferroni’s correction). In this figure, statistics are displayed as: * p<0.05, ** p<0.01, *** p<0.001. (TIF) [file pgen.1006695.s009.tif]

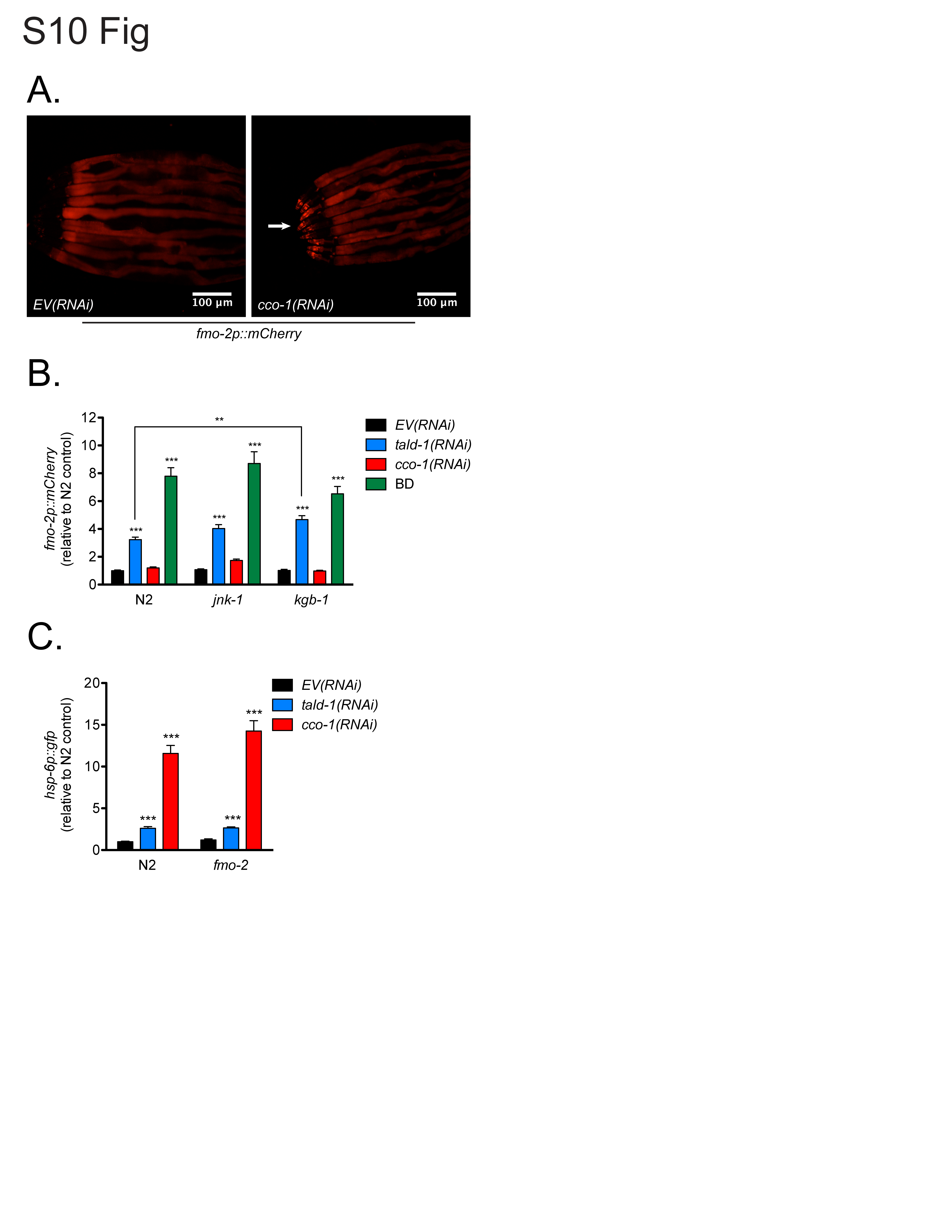

Supplement: S10 Fig — (A) RNAi knockdown of cco-1 increases fmo-2p::mCherry reporter induction in cells proximal to the anterior bulb. fmo-2p::mCherry reporter animals were grown on RNAi bacteria from hatching and imaged 4 days later using fluorescent microscopy. (B) Mean relative fluorescence of fmo-2p::mCherry reporter animals in the context of jnk-1(gk7) and kgb-1(um3) mutations. Fluorescence is calculated relative to N2 EV(RNAi) controls (N = 3 independent experiments, pooled individual worm values, error bars indicate s.e.m., ANOVA with Bonferroni’s post-hoc). (C) Mean relative fluorescence of hsp-6p::gfp reporter animals in the context of the fmo-2(ok2147) mutation. Fluorescence is calculated relative to N2 EV(RNAi) controls (N = 3 independent experiments, pooled individual worm values, error bars indicate s.e.m., student’s t-test with Bonferroni’s correction). In this figure, statistics are displayed as: * p<0.05, ** p<0.01, *** p<0.001. (TIF) [file pgen.1006695.s010.tif]
